# Synthesis of unprotected thienyl sulfonamides and their activities against carbapenem-resistant *Klebsiella pneumoniae*, docking studies and ADMET analysis

**DOI:** 10.1039/d5ra05409e

**Published:** 2025-12-03

**Authors:** Muhammad Bilal, Mnaza Noreen, Muhammad Usman Qamar, Farhan Siddique, Nasir Rasool, Muhammad Imran

**Affiliations:** a Department of Chemistry, Government College University Faisalabad 38000 Pakistan nasirrasool@gcuf.edu.pk; b Institute of Microbiology, Faculty of Life Sciences, Government College University Faisalabad 38000 Faisalabad Pakistan; c Department of Pharmaceutical Chemistry, Faculty of Pharmacy, Bahauddin Zakariya University 60800 Multan Pakistan; d Chemistry Department, Faculty of Science, King Khalid University P. O. Box 9004 Abha 61413 Saudi Arabia

## Abstract

Carbapenem-resistant *Klebsiella pneumoniae* (CRKP), in particular hypervirulent and classical strains, represents a severe global health burden with limited treatment options. The urgent need for new antimicrobials motivates the exploration of novel chemical scaffolds. This study focused on substituted thiophene-based thienyl sulfonamides, synthesized *via* the Suzuki–Miyaura cross-coupling with moderate to excellent yields of unprotected compounds. Evaluation against clinical CRKP isolates revealed significant antibacterial activity for several synthesized sulfonamides. Molecular docking and ADMET profiling further identified compounds 3c, 3f, and 3g as possessing potent activity, promising binding characteristics, and suitable pharmacological properties. These results highlight these thienyl sulfonamides as viable lead candidates for combating multidrug-resistant *K. pneumoniae* infections.

## Abstract 2

Substituted thiophene-based thienyl sulfonamides exhibit potent antibacterial activity against clinical CRKP isolates. Docking and ADMET studies identified compounds 3c, 3f, and 3g as promising lead candidates with favorable pharmacological profiles.

## Graphical abstract

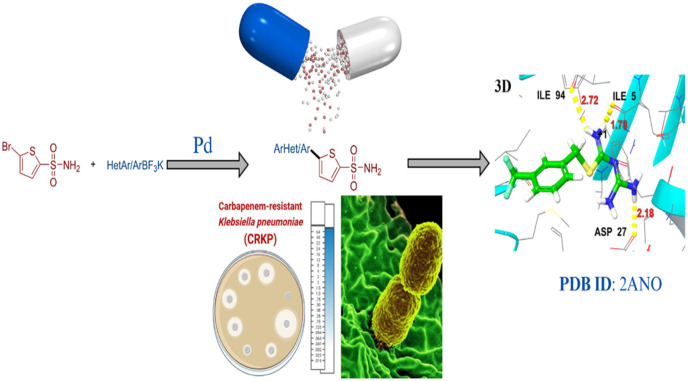

## 1. Introduction

Pathogenicity patterns differentiate classical strains from hypervirulent carbapenem-resistant *K. pneumoniae* (CRKP) strains. Classical strains characteristically result in hospital-acquired urinary tract infections, primarily impacting immunocompromised and elderly hosts. Hypervirulent strains, however, are notable for inducing severe invasive diseases with substantial morbidity, capable of infecting both immunologically intact individuals and those with compromised immunity.^1–3^ Carbapenem antibiotics represent the cornerstone therapeutic agents for severe infections caused by multidrug-resistant (MDR) Enterobacterales.^4^ The broad-spectrum activity of carbapenems is derived from their distinctive β-lactam ring structure, which confers stability against hydrolysis by diverse β-lactamases including extended-spectrum β-lactamases (ESBLs) and metallo-β-lactamases (MBLs).^5^

**Fig. 1:**
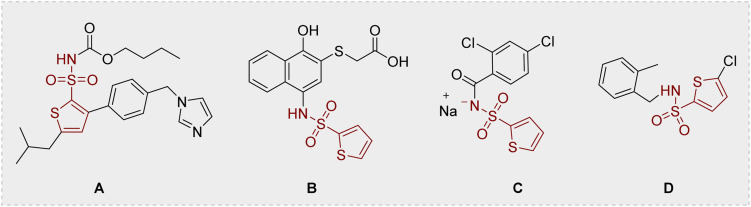
Chemical structures of the biorelevant thienyl sulfonamides.

Resistance to carbapenems raises a paramount global health concern, primarily because they remain the ultimate therapeutic option for severe infections caused by bacterial strains exhibiting resistance to nearly all other antimicrobial classes.^6–10^ CRKP infections commonly result in poor clinical outcomes, including high mortality rates, given the limited therapeutic options available, cementing its position as a major global public health priority.^11,12^ Carbapenemase production (primarily mediated by genes such as bla_NDM-1_ or bla_KPC-2/3_) combined with the expansion of epidemic clones (*e.g.*, ST258/512) fuels the global spread of CRKP, while substantial intercontinental differences govern its regional evolutionary trajectories and clinical strain distributions.^13^ In Asia, particularly China, bla_KPC-2_-producing *K. pneumoniae* ST11 is the dominant CRKP lineage accounting for 60–70% of clinical isolates.^14^ Evolved from ST11, the ST258 lineage dominates CRKP epidemiology in non-Asian regions, serving as the principal carrier of KPC-2/KPC-3 carbapenemases throughout the Americas and Europe.^15,16^ Despite maintaining high regional prevalence, extensive genetic diversification has occurred within pandemic clones ST11 and ST258 *via* intra-clonal segregation driven primarily by recombination events in the capsular polysaccharide synthesis locus. These events catalyze the emergence of antigenically distinct subclones.^17,18^ An intra-clonal transition from ST11-KL47 to ST11-KL64 occurred among bacteremic CRKP isolates in China (single center, 2013–2017). The emergent KL64 subclone demonstrates enhanced virulence, conferring an elevated 30-day mortality risk. Spatiotemporal dynamics and selective pressures driving this displacement are incompletely characterized.^19–21^ The global CRKP crisis demands urgent development of novel antimicrobials, propelled by the near-total absence of effective therapies against dominant ST258/ST11 lineages expressing KPC enzymes. Substituted thiophenes represent privileged scaffolds in drug discovery, widely exploited as versatile heterocyclic cores for designing bioactive molecules with tailored pharmacological profiles.^22–30^ Thienyl sulfonamides have been of particular interest in medicinal chemistry and are abundant in many biologically active compounds.^31^ The discovery of thienyl sulfonamide (A) in 2004 marked the first identification of a drug-like compound exhibiting selective agonist activity (M024/C21) at the AT2 receptor.^32^ Lawrence's team reported a thienyl sulfonamide group containing proteasome inhibitors (B).^33^ Waters *et al.* discovered that substituted thienyl sulfonamides act as inhibitors targeting both malarial and mammalian cyclin-dependent kinases (CDKs).^34^ Tasisulam (LY573636·Na, C) represents a novel anticancer agent characterized by cytotoxicity and the ability to induce apoptosis.^35^ Recently, the Rajashekara group has reported that the benzyl thiophene sulfonamide derivatives (D) are effective against Campylobacter^36^ (Fig. 1).

Sulfonamide antibiotics have remained clinically important since their introduction in 1968. In primary care settings, they serve as first-line treatments for upper urinary and respiratory tract infections due to their favorable tolerability and low cost.^37^ Beyond their antibiotic role, sulfonamide derivatives function as antibacterial and antiviral agents in chemotherapy regimens.^38–40^ Therapeutically, these compounds exploit structural similarity to 4-aminobenzoic acid (PABA) to act as competitive antagonists. They specifically target bacterial folate biosynthesis by inhibiting PABA incorporation during folate synthase catalysis. This disruption prevents bacterial folic acid synthesis, crippling essential purine production.^41–44^ Recently, we have found that 5-bromo-*N*-alkylthiophene-2-sulfonamides displayed antibacterial efficacy against NDM-β-lactamase-producing *K. pneumoniae* ST147.^45^

Herein, we report the synthesis of unprotected thienyl sulfonamides *via* the Suzuki–Miyaura cross-coupling reaction in moderate to excellent yields. Then we investigated *in vitro* anti-bacterial activities against clinically isolated carbapenem-resistant *K. pneumoniae* (CRKP), which were further validated by docking studies and ADMET analysis.^46^ We targeted the DHFR enzyme to exploit a pathway essential for bacterial survival but equally distinct from β-lactam resistance in CRKP. This well-characterized active site aided in the structure-based design of our thienyl sulfonamides, and incorporated docking studies guided the development of these novel potential inhibitors.

## 2. Result and discussion

### 2.1. Chemistry

**Scheme 1:**
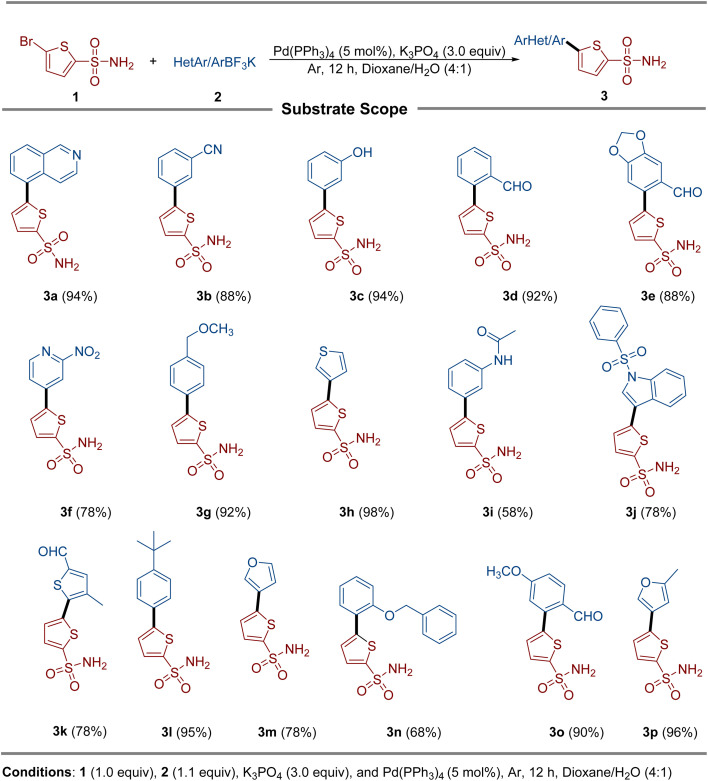
Synthesis of the thienyl sulfonamide derivatives.

A library of unprotected thienyl sulfonamide derivatives (3) were synthesized *via* the Suzuki–Miyaura cross-coupling reaction between 5-bromothiophene-2-sulfonamide (1) and diverse aryl/heteroaryltrifluoroborates (2). This method achieved moderate to excellent yields across a broad substrate scope, demonstrating remarkable functional group tolerance. Electron-donating groups and strongly electron-withdrawing substituents were fully compatible, consistently affording excellent yields. Chloride, nitrile, and aldehyde functionalities remained intact under the reaction conditions, enabling access to synthetically versatile intermediates. Notably, the amide-substituted trifluoroborate (3i) yielded poorly, probably due to the hydrolytic cleavage of the amide bond under basic cross-coupling conditions. The robustness of this protocol is highlighted by its tolerance of aldehydes, a functionality traditionally incompatible with organoboron chemistry due to competitive formylation or protodeboronation. Similarly, the nitro group and hydroxyl group posed no observable reactivity issues (Scheme 1).

### 2.2. Identification of the isolate

The isolate exhibited antibiotic resistance from the WHO's Access, Watch, and Reserve (AWaRe) categories. The MICs of ampicillin (≥32 g L^−1^) and amoxicillin/clavulanic acid (≥32/16 g L^−1^) were higher than normal. However, ciprofloxacin (≥1 g L^−1^) was the most effective medication with the highest sensitivity against the isolate (Table 1). Molecular identification of the blaNDM-1 gene showed that carbapenem-resistant *K. pneumoniae* carried blaNDM-1.

**Table 1:** MICs of the antibiotics against carbapenem-resistant *K. pneumoniae*^a^

Antibiotics	MIC (g L^−1^) breakpoints	MIC value
AMP	≥32	≥128
AMC	≥32/16	≥128/64
CRO	≥4	≥128
FEP	≥16	≥64
CAZ	≥16	≥128
IMP	≥4	≥16
MEM	≥4	≥16
CIP	≥1	≥16
AK	≥16	8
TE	≥16	≥64
TGC	≥8	4
TMP	≥16	≥64
CS	≥4	≤0.5

a AMP: ampicillin; AMC: amoxicillin/clavulanic acid; CRO: ceftriaxone; FEP: cefepime; CAZ: ceftazidime; IMP: imipenem; MEM: meropenem; CIP: ciprofloxacin; AK: amikacin; TE: tetracycline; TGC: tigecycline; TMP: trimethoprim; and CS: colistin.

### 2.3. Biological activities

The anti-bacterial activity of the molecules 3a–3p was evaluated against carbapenem-resistant *Klebsiella pneumoniae* by the agar well diffusion method at five different concentrations (10, 20, 30, 40, and 50 mg per well). The results demonstrated that compounds 3c, 3f, and 3g exhibited the lowest MIC and MBC values of 31.25 µg mL^−1^ and 62.5 µg mL^−1^, respectively. In comparison, compounds 3a, 3b, and 3o displayed an MIC of 62.5 µg mL^−1^ and an MBC of 125 µg mL^−1^, as presented in Table 2. The results show that the compounds 3c, 3f, and 3g are the effective candidates among the synthesized compounds, while 3a, 3b, and 3o show moderate activities. The other compounds are almost inactive against the CRKP.

**Table 2:** Antibacterial activity of the compounds against carbapenem-resistant *K. pneumoniae*

Compounds	MIC (µg mL^−1^)	MBC (µg mL^−1^)
3a (1)	62.5	125
3b (3)	62.5	125
3c (4)	31.25	62.5
3d (6)	125	250
3e (7)	250	500
3f (8)	31.25	62.5
3g (15)	31.25	62.5
3h (17)	125	250
3i (18)	250	500
3j (20)	125	250
3k (21)	250	500
3l (23)	250	500
3m (25)	125	250
3n (26)	250	500
3o (27)	62.5	125
3p (28)	250	500

### 2.4. Docking studies

**Fig. 2:**
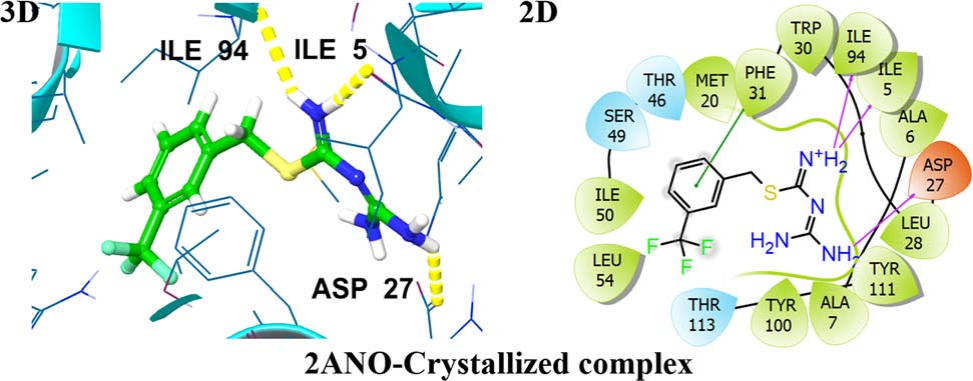
Representation of 3D and 2D interactions of the co-crystallized ligands with DHFR (PDB ID: 2ANO).

A validated molecular docking method was utilized to explore the inhibiting potential of bioactive compounds towards DHFR (PDB ID: 2ANO) at the molecular level (Fig. 2). As a key step in confirming the accuracy of molecular docking, the co-crystallized ligand was re-docked into the binding site of the target protein. The docking results demonstrated a remarkably low root mean square deviation (RMSD) of about 1.2 Å when compared to the original conformation, underscoring the precision and reliability of the docking protocol. Subsequent interaction analysis of crystallized ligand revealed robust binding engagement, forming three hydrogen bonds with residues ILE5, ASP27, and ILE94. These interactions contributed to its favorable docking score of −7.79 kcal mol^−1^. The 3D and 2D interactions of a co-crystallized ligand with the target protein are visualized in Fig. 2.

**Fig. 3:**
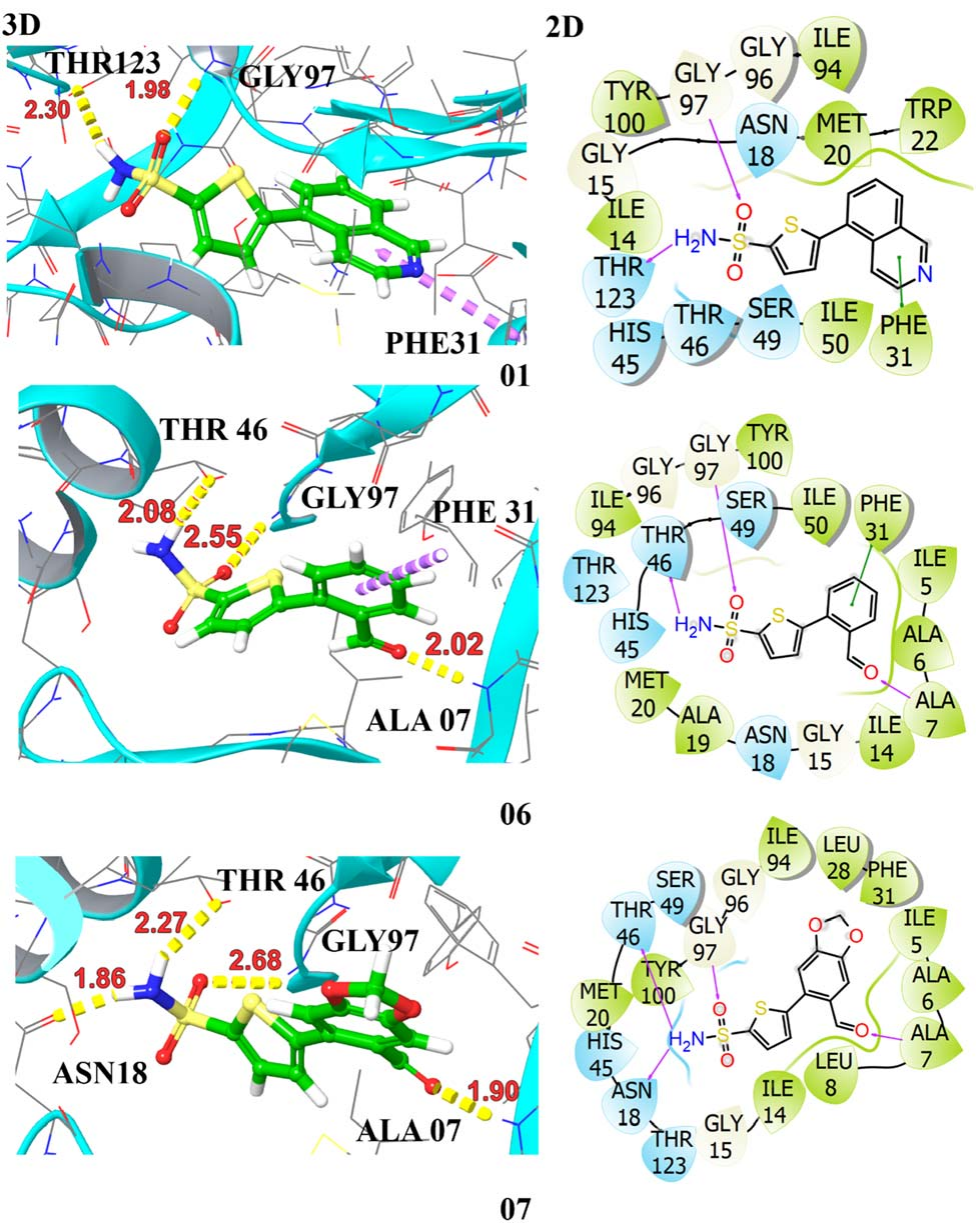
Illustration of the 3D and 2D interactions of the top hits compounds 3a (1), 3d (6), and 3e (7) with the target protein DHFR (PDB ID: 2ANO).

**Fig. 4:**
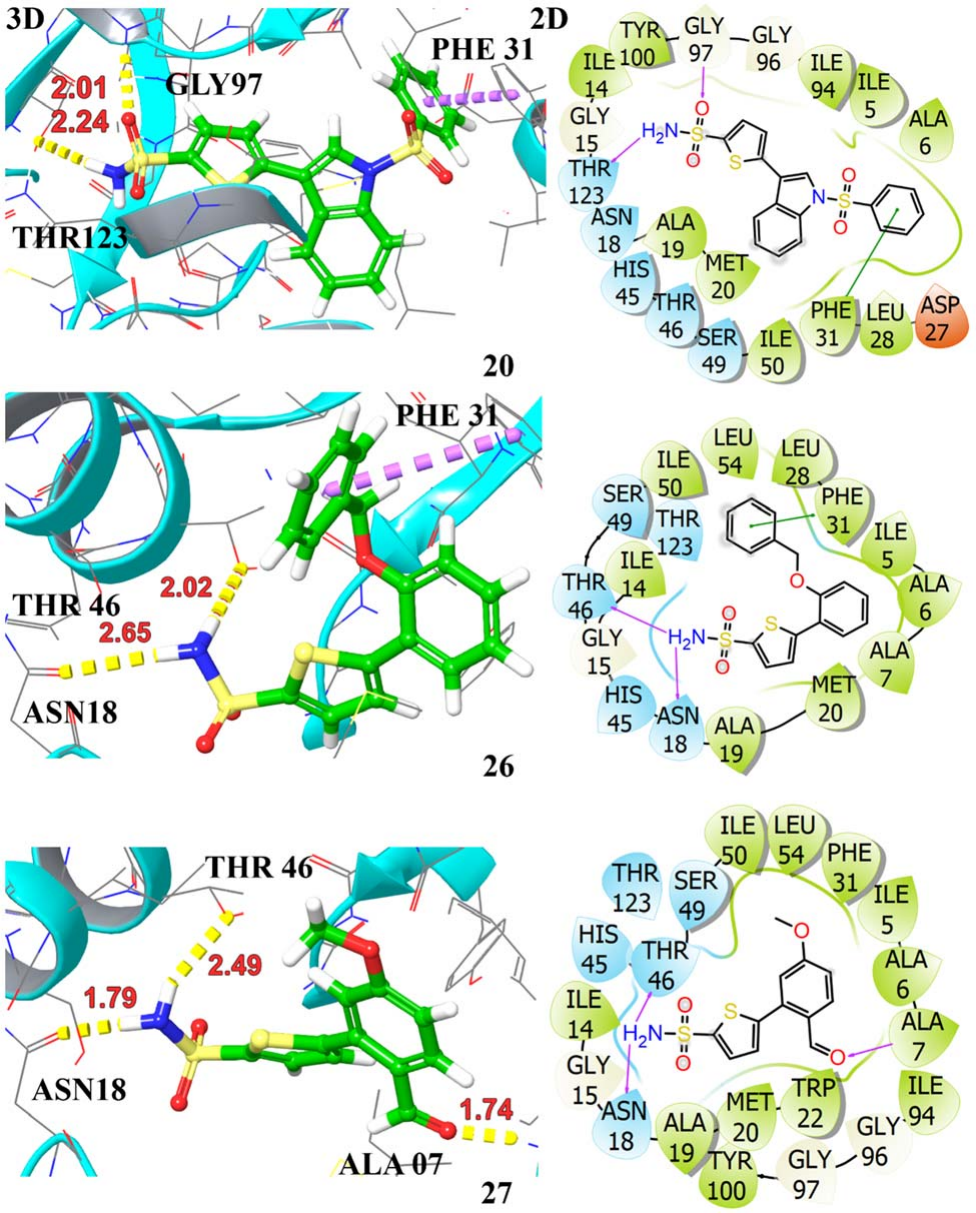
Illustration of the 3D and 2D interactions of the top hit compounds 3j (20), 3n (26), and 3o (27) with the target protein DHFR (PDB ID: 2ANO).

The docking profiles including *G*-scores and E-model scores of 16 compounds along with key binding residues involved in H-bonding and hydrophobic are systematically summarized in Table 3. Molecular docking of the studied compounds led to the identification of six promising candidates, namely compounds 3a, 3d, 3e, 3j, 3n, and 3o, demonstrating substantial binding affinities with docking scores >−7.0 kcal mol^−1^ against the target protein. The compounds 3d and 3e exhibited H-bonding interactions with ALA07, ASN18, GLY97, and THR46, complemented by aromatic π–π interaction with PHE31. These interactions contributed to a significantly higher affinity with binding energies of −8.42 kcal mol^−1^ and −8.11 kcal mol^−1^, respectively. Compound 3o achieved a docking score of −7.86 kcal mol^−1^, demonstrating slightly higher affinity in comparison to the crystallized compound. It established hydrogen bonds with ALA07, ASN18, and THR46, attributed to its strong anchoring within the enzyme's active site. The compounds 3a, 3j, and 3n also showed potent binding against the target protein, securing a docking score of −7.42, −7.36, and −7.64 kcal mol^−1^, respectively. They demonstrated two H-bonding interactions with key amino acid residues, stabilized within the binding pocket *via* π–π stacking with PHE31, indicative of their significant inhibiting potential towards the target protein. The least binding affinity is observed for compound 3i with a *G*-score of −5.90 kcal mol^−1^, demonstrating only π–π stacking with PHE31. The 3D and 2D interactions of top hits are illustrated in Fig. 3 and 4. The potent compounds 3c, 3f, and 3g also show significant docking scores of −6.45, −6.45, and −6.19, respectively. These insights provide a compelling rationale for further experimental validation and development of these compounds, particularly to combat antibiotic resistance.

**Table 3:** Molecular Glide score, hydrogen bonding, and hydrophobic and other interactions with distances (Å) for the top hit compounds with DHFR (PDB ID: 2ANO)^a^

Sr. no.	Compounds name (code)	*G*-score (kcal mol^−1^)	Emodel	H.B.I residue (distance measured in Å)	Hydrophobic and other interacting residues
1	3a (01)	−7.42	−60.62	THR123 (2.30)	PHE31
				GLY97 (1.98)	
2	3b (03)	−6.09	−52.90	ILE14 (2.55)	Not found
				ASN18 (2.22)	
				THR46 (2.17)	
				GLY97 (2.77)	
3	3c (04)	−6.45	−52.93	ILE14 (2.65)	PHE31
				ASN18 (2.24)	
				THR46 (2.17)	
				GLY97 (2.59)	
4	ILE94 (2.72) (06)	−8.42	−62.87	THR46 (2.08)	PHE31
				GLY97 (2.55)	
				ALA07 (2.02)	
5	3e (07)	−8.11	−62.75	ALA07 (1.90)	Not found
				ASN18 (1.86)	
				THR46 (2.27)	
				GLY97 (2.68)	
6	3f (08)	−6.47	−58.79	ALA07 (2.62)	Not found
				ILE 14 (2.19)	
				GLY97 (2.03)	
				THR123 (2.45)	
7	3g (15)	−6.91	−60.59	ASN18 (2.26)	Not found
				THR46 (2.14)	
				GLY97 (2.48)	
8	3h (17)	−6.33	−48.31	ILE94 (2.51)	PHE31
				ILE94 (2.54)	
9	3i (18)	−5.90	−48.94	Not found	PHE31
10	3j (20)	−7.36	−72.74	THR123 (2.24)	PHE31
				GLY97 (2.01)	
11	3k (21)	−6.41	−54.18	ILE94 (2.48)	Not found
				PHE31 (3.18)	
12	3l (23)	−6.10	−51.57	ASN18 (2.49)	Not found
				SER49 (2.68)	
				GLY97 (2.38)	
				THR123 (2.43)	
13	3m (25)	−6.42	−45.79	ILE14 (2.53)	Not found
				ASN18 (1.98)	
				THR46 (2.09)	
				GLY97 (2.65)	
14	3n (26)	−7.64	−68.02	ASN18 (2.65)	PHE31
				THR46 (2.02)	
15	3o (27)	−7.86	−63.19	ALA07 (1.74)	Not found
				ASN18 (1.79)	
				THR46 (2.49)	
16	3p (28)	−6.10	−49.05	ASN18 (2.28)	Not found
				THR46 (2.09)	
				GLY97 (2.62)	
				GLY97 (2.64)	
CL	MS-SH08-17 (1-{[*N*-(1-imino-guanidino-methyl)]sulphanylmethyl}-3-trifluoromethyl-benzene)	−7.79	−62.43	ILE05 (1.78)	ILE5, ALA6, ALA7, ALA19, MET20, LEU28, TRP30, PHE31, THR46, SER49, ILE50, LEU54, TYR100, THR113
				ASP27 (2.18)	
				ILE94 (2.72)	

a CL: crystallized ligand and H.B.I: hydrogen bonding interacting residues.

The computed docking studies successfully forecasted the binding poses of the synthesized derivatives within the active binding site of the target receptor enzyme. On the flip side of it, an expected direct correlation between the computed docking scores (ranging from −8.42 to −6.50 kcal mol^−1^) and the experimental bioactivity was not observed. As seen in the case of compound 3d, which exhibited one of the most favorable docking scores (−8.42 kcal mol^−1^), it showed only a modest activity, while highly active compounds such as 3c and 3g had more modest scores (*e.g.*, −7.21 and −7.32 kcal mol^−1^, respectively). The highlighted discrepancy thus suggests that factors beyond the observed simple binding affinity are critical determinants of the experimental cytotoxicity. This discrepancy shows the critical significance of employing integrated ADMET outcomes with structure-based designing, because the bioactivity is not solely reliant on the feature of binding affinity, but it is a function of a compound's overall pharmacokinetic profile. Basically, molecular docking is a snapshot of a static binding event.

### 2.5. ADMET results

Table 4 lists the pharmacokinetic properties and drug likeness of the studied compounds, calculated by employing SwissADME. The log *P* value was predicted using five distinct models, namely, XLOGP3, WLOGP, MLOGP, SILICOS-IT, and iLOGP, culminating in a consensus log *P* value. Most of the compounds demonstrated optimum XLOGP3 and WLOGP values within the range of 1–3 and 2–4, respectively, representing their significant lipid solubility. Compounds 3j, 3l, and 3n slightly exceed the upper limit of optimal XLOGP3 and WLOGP values. Additionally, topological polar surface area (TPSA) was calculated as a key determinant of permeability, representing that compounds with a TPSA below 140 Å^2^ generally showed good oral bioavailability, while those below 90 Å^2^ were more likely to cross the blood–brain barrier (BBB).^47^ Most of the studied compounds demonstrated TPSA values within the range of 96.78 Å^2^ to 132.31 Å^2^, representing their optimal pharmacokinetic properties. The compounds 3f, 3j, and 3k exceed the upper limit of TPSA, demonstrating their low GI absorption. *In silico* ADMET profiling indicates that the potent compound 3c exhibits excellent drug-like properties, including high predicted GI absorption and favorable permeability. On the contrary, the compound with the best docking score (3d, *G* score = −8.42 kcal mol^−1^) reflects the poor predicted passive permeability (log *K*_p_ = −6.78 cm s^−1^) among its investigated peers, which likely impedes its cellular uptake and thus clearly explains its lower than expected experimental activity.

**Table 4:** The pharmacokinetic properties and drug likeness of the studied compounds were calculated using the SwissADME database^a^

Compounds	TPSA	XLOGP3	WLOGP	GI absorption	BBB permeant	Pgp substrate	CYP2D6 inhibitor	Log *K*_p_ (cm s^−1^)	Lipinski #violations
3a (1)	109.67	2.34	3.69	High	No	No	Yes	−6.41	0
3b (3)	120.57	1.88	3.01	High	No	No	No	−6.58	0
3c (4)	117.01	1.81	2.85	High	No	No	No	−6.57	0
3d (6)	113.85	1.62	2.96	High	No	No	No	−6.78	0
3e (7)	132.31	1.44	2.68	High	No	No	No	−7.18	0
3f (8)	155.49	1.25	2.45	Low	No	No	No	−7.15	0
3g (15)	106.01	1.81	3.14	High	No	No	No	−6.74	0
3h (17)	125.02	1.84	3.2	High	No	No	No	−6.49	0
3i (18)	125.88	1.34	2.91	High	No	No	No	−7.16	0
3j (20)	144.23	3.47	5.42	Low	No	No	No	−6.39	0
3k (21)	142.09	2.04	3.33	Low	No	No	No	−6.6	0
3l (23)	96.78	3.83	4.44	High	No	No	No	−5.38	0
3m (25)	109.92	1.23	2.74	High	No	No	No	−6.83	0
3n (26)	106.01	3.63	4.57	High	No	No	Yes	−5.83	0
3o (27)	123.08	1.6	2.96	High	No	No	No	−6.98	0
3p (28)	109.92	1.63	3.04	High	No	No	No	−6.63	0

a TPSA: topological polar surface area, GI: gastrointestinal BBB: blood–brain barrier, and Pgp: P-glycoprotein.

**Fig. 5:**
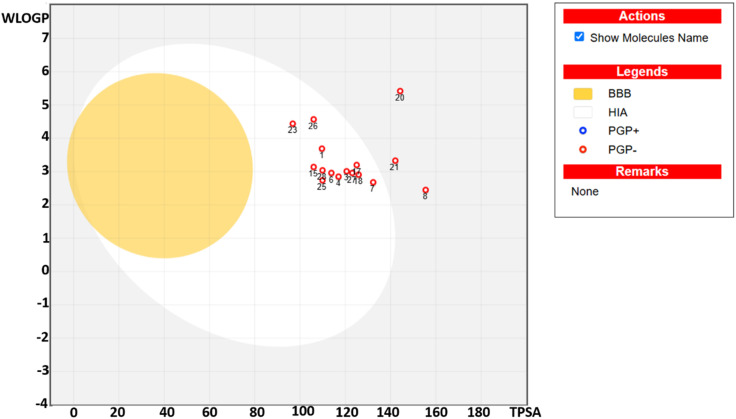
The boiled-egg presentation of the studied compounds.

Pharmacokinetic profiling using the BOILED-Egg model (Fig. 5) highlighted the GI absorption and potential BBB permeability of the compounds. The compounds studied are not shown in the yellow region, indicating their inability to cross the BBB and thus being devoid of CNS toxicity. The compounds in the white region indicate their significant intestinal absorption, whereas compounds 3f, 3j and 3k in the grey region illustrate their poor GI absorption. The compounds studied were not found to be the substrate of P-glycoprotein, representing their significant bioavailability. The studied compounds do not interact with cytochrome P450 (CYP2D6), aiding in the assessment of their metabolic stability. Moreover, drug-likeness was assessed using Lipinski's rules of five to prioritize compounds with favorable pharmacokinetic and safety profiles. Collectively, these silico analyses support the potential of the sixteen compounds as promising drug candidates.

## 3. Methodologies

### 3.1. Synthesis of thienyl sulfonamide derivatives (3a–3p)

To a Schlenk tube containing 5-bromothiophene-2-sulfonamide (1.0 equiv., 1.00 mmol), aryl/heteroaryltrifluoroborate (1.1 equiv., 1.1 mmol), potassium phosphate (K_3_PO_4_, 3.0 equiv., 3.0 mmol), and tetrakis(triphenylphosphine)palladium(0) [Pd(PPh_3_)_4_, 5 mol%] were added. The vial was purged with argon three times to ensure an inert atmosphere. Subsequently, 1,4-dioxane and water (4 : 1) were added to the reaction mixture. The mixture was stirred at 100 °C, and the reaction progress was monitored by TLC. Upon completion, the reaction was cooled to room temperature and extracted with ethyl acetate. The combined organic layers were filtered, dried over anhydrous sodium sulfate, and concentrated under reduced pressure. The resulting crude product was purified by flash column chromatography using a gradient of ethyl acetate and hexane to afford the desired compound. The final product was characterized using standard spectroscopic techniques.

### 3.2. Identification and isolation of bacterial strain

The complete procedure is mentioned in the SI.

### 3.3. Anti-bacterial activities

#### 3.3.1. Minimum inhibitory concentration of different compounds against CRKP

The minimum inhibitory concentration (MIC) of each compound was determined by the micro broth dilution method, as described previously.^48^ For bacterial culture preparation, 20 mL of double-strength lysogeny broth (LB) was inoculated with two to three well-isolated colonies in a 50 mL Falcon tube and incubated at 37 °C for 24 hours. The resulting bacterial suspension was then diluted to an optical density (OD_600_) of 0.07, corresponding to a 0.5 McFarland turbidity standard. For the MIC assay, serial dilutions of each test compound were prepared in dimethyl sulfoxide (DMSO) at concentrations of 0.76, 1.56, 3.12, 6.25, 12.5, 25, and 50 µg mL^−1^. A 96-well flat-bottom microtiter plate was then set up, with 100 µL of each compound dilution added to the respective wells. Subsequently, 100 µL of the bacterial suspension was added to each well, yielding a final volume of 200 µL per well. Negative control wells contained 100 µL of LB only, while positive controls included both LB and bacterial suspension. The plate was incubated overnight at 37 °C in a shaking incubator (MaxQ™ Mini 4450, Thermo Fisher Scientific). The MIC values were determined by the visual comparison of bacterial growth in test wells relative to the positive and negative controls. All experiments were conducted in triplicate to ensure reproducibility and accuracy. In the microtiter plate, column no. 11 is the NC (negative control), which includes only the Mueller Hinton broth with no bacterial isolates; however, column no. 12 is the PC (positive control) containing the bacterial growth.

#### 3.3.2. Minimum bactericidal concentration against CRKP

The minimum bactericidal concentration (MBC) was identified as the lowest concentration of the compound that prevented visible bacterial growth on nutrient agar. From each well in the microtiter plate that showed no visible turbidity, a 10 µL aliquot was aseptically transferred and streaked into nutrient agar plates. The plates were then incubated aerobically at 37 °C for 24 hours. Following incubation, the plates were examined for bacterial growth to assess cell viability. The absence of visible colonies indicated bactericidal activity at that concentration. All experiments were conducted in triplicate to ensure the reproducibility and accuracy of the results.

### 3.4. Molecular docking studies

Molecular docking studies were executed using the Glide module of the Schrödinger Suite (version 2019-1)^49^ to investigate the antibacterial potential *via* binding interactions of the most pharmacologically active compound with the target protein. The 3D structure of the dihydrofolate reductase (PDB ID: 2ANO)^50,51^ was retrieved from the RCSB Protein Data Bank (https://www.rcsb.org/structure/2ANO). Protein preparation was carried out using Schrödinger's Protein Preparation Wizard, involving the addition of hydrogen atoms, adjustment of bond orders, and prediction of ionization states using Epik at physiological pH (7.0 ± 2.0). The ligands were initially sketched using ChemDraw 18.0 (ref. 52) and were converted into 3D formats. The structures were energetically optimized using LigPrep with the OPLS4 force field, which ensured accurate geometry, bond assignments, and the addition of hydrogen atoms. A receptor grid was generated by centering on the co-crystallized ligand, with the van der Waals radii scaled to 1.00 Å and partial charges set at 0.25, defining the active site environment.^53^ To validate the docking protocol, the native ligand was redocked into the protein's active site, resulting in a root mean square deviation (RMSD) of 0.20 Å, which confirms the protocol's precision and reliability. Following validation, docking simulations were performed in extra precision (XP) mode, applying a van der Waals scaling factor of 0.8 and a charge cutoff of 0.15 to enhance the accuracy. The resulting docked conformations were ranked based on Glide scores, and interaction profiles were evaluated using the XP Visualizer tool.^49,54^

### 3.5. *In silico* ADMET

The SwissADME web tool, developed by the Swiss Institute of Bioinformatics (https://www.sib.swiss/), was utilized to predict the ADME (Absorption, Distribution, Metabolism, and Excretion) profiles of studied compounds.^55^

## 4. Conclusion

A series of unprotected thienyl sulfonamide derivatives (3a–3p) were synthesized by the Suzuki–Miyaura cross-coupling, which were further assessed against the clinically isolated multidrug-resistant carbapenem-resistant *Klebsiella pneumoniae*. The compounds 3c, 3f, and 3g demonstrated significant antibacterial activity. These compounds have an MIC value of 31.25 µg mL^−1^ and an MBC value of 62.5 µg mL^−1^. This study employed molecular docking to explore the anti-bacterial potential of the studied compounds with the target protein (DHFR). The docking results revealed significant binding affinities of compounds 01, 06, 07, 20, 26, and 27 with *G*-scores >−7.0 kcal mol^−1^. These top hits formed critical interactions such as hydrogen bonds, hydrophobic contacts, and π–π stacking, suggesting their potential as lead compounds for antibacterial drug development. *In silico* ADMET profiling of sixteen compounds revealed favorable lipophilicity and pharmacokinetic properties, with most falling within drug-like thresholds for XLOGP, WLOGP, and TPSA, with zero violation of Lipinski rules of five.

## Conflicts of interest

There are no conflicts of interest to declare.

## Supplementary Material

RA-015-D5RA05409E-s001

## Data Availability

Spectroscopic data for the compounds (^1^H NMR, ^13^C NMR) and the methods used for bacterial isolation and identification are provided in the supplementary information (SI) or are available from the authors upon request. Supplementary information is available. See DOI: https://doi.org/10.1039/d5ra05409e.
