# Supplementary material for: Synthesis of unprotected thienyl sulfonamides and their activities against carbapenem-resistant Klebsiella pneumoniae, docking studies and ADMET analysis
Source: RSC Adv. 2025 Dec 3;15(55):47700–9. doi: 10.1039/d5ra05409e (PMC12679364; doi:10.1039/d5ra05409e)
Supplement: RA-015-D5RA05409E-s001 [file RA-015-D5RA05409E-s001.pdf]

Supplementary Information (SI) for RSC Advances.
This journal is © The Royal Society of Chemistry 2025

Synthesis of unprotected thienyl sulfonamides and its activities against Carbapenem-resistant *Klebsiella pneumoniae*, docking studies and ADMET analysis

Muhammad Bilal[1,#], Mnaza Noreen[1,#], Muhammad Usman Qamar[2], Farhan Siddique[3], Nasir Rasool[1,*], Muhammad Imran[4]

[1]Department of Chemistry, Government College University Faisalabad, 38000, Pakistan

[2] Institute of Microbiology, Faculty of Life Sciences, Government College University Faisalabad 38000, Faisalabad.

[3]Department of Pharmaceutical Chemistry, Faculty of Pharmacy, Bahauddin Zakariya University 60800, Multan. Pakistan

[4]Chemistry Department, Faculty of Science, King Khalid University, P.O. Box 9004, Abha 61413, Saudi Arabia

*Correspondence: nasirrasool@gcuf.edu.pk

Table of Contents

1. Identification and isolation of bacterial strain....................3

2. MIC against CRKP....................4

3. Spectroscopic data....................4

4. References....................6

5. NMR spectra for products....................6

1. Identification and isolation of bacterial strain

Ethical approval from the Ethical Review Board of Government College University, Faisalabad, and informed parental consent, compliant with the Declaration of Helsinki, preceded the collection of a 3 mL blood sample from a 5-year-old pediatric patient suspected of septicemia in tertiary care. This blood was added to a BACTEC/Alert Pediatric vial. Using the BACTEC/Alert automated system (BD, UK), the vial was incubated at 37 ºC for a maximum of 5 days. Upon positivity, subculture occurred on blood agar and MacConkey agar (Oxoid, UK), with overnight aerobic incubation at 37 ºC. The resultant isolate underwent primary identification via colony characteristics, followed by biochemical confirmation via a Gram-negative card and the VITEK 2 compact system (Biomerieux, France).

1.1. Sequence Typing of K. Pneumoniae

The selected housekeeping genes for *K. pneumoniae* sequence typing included RNA polymerase β-subunit (rpoB), glyceraldehyde-3-phosphate dehydrogenase (gapA), translation initiation factor 2 (infB), malate dehydrogenase (mdh), phosphoglucose isomerase (pgi), phosphorene E (phoE), and periplasmic energy transducer (tonB). Following agarose gel extraction *via* QIAquick Kit (Qiagen, Germany), amplicons were outsourced for DNA sequencing to Eurofins Scientific (London). The obtained sequencing data were then analyzed using the *K. pneumoniae* MLST database available at http://bigsdb.pasteur.fr/klebsiella/klebsiella.html.

1.2. Antibiogram of the Isolate

Antimicrobial susceptibility was assessed using the VITEK 2 compact automated system (BioMerieux, France) to derive MIC values for relevant antibiotics. MICs are quantified in μg/mL units. The antimicrobials tested were ampicillin/sulbactam, piperacillin, levofloxacin, cefepime, aztreonam, ceftriaxone, amikacin, minocycline, meropenem, moxifloxacin, tetracycline, colistin, and chloramphenicol. Susceptibility was interpreted according to the following CLSI 2021 recommendations [1].

1.3. Phenotypic Detection of Carbapenemase Enzyme

Carbapenemase activity was assessed via the Modified Hodge Test (MHT) [2]. For the Modified Hodge Test (MHT), bacterial isolates were streaked radially on Mueller-Hinton agar (MHA) plates. A meropenem disc was placed centrally, and plates were incubated at 35±2 ºC for 18-24 hours. Carbapenemase production was confirmed by a characteristic cloverleaf-shaped indentation of bacterial growth toward the meropenem disc. Metallo-β-lactamase (MBL) production was detected using a double-disc synergy test. A confluent lawn of *K. pneumoniae* was prepared on MHA. Discs containing imipenem and meropenem were placed 20 mm apart, with 10 µL of 0.5 M EDTA applied directly to each carbapenem disc. A positive MBL result was defined as a ≥5 mm increase in the zone of inhibition around the EDTA-supplemented disc compared to the carbapenem disc alone [3].

1.4. Phenotypic Detection of Metallo-β-Lactamase (MBL) Enzyme

MBL-producing bacteria were identified using the double-disc synergy method, incorporating EDTA. Briefly, the isolates were spread onto Mueller-Hinton agar (MHA) plates, and two discs containing imipenem and meropenem were positioned 24 mm apart. Subsequently, 10 µL of a 0.5M EDTA solution was applied to each carbapenem disc. A positive result was indicated if the EDTA discs displayed a zone of inhibition greater than 5 mm compared to the non-EDTA discs [3].

1.5. Molecular Characterization of bla_{NDM}

The extraction of DNA from bacterial culture was carried out using a DNA kit for bacterial cells (TIANamp, Beijing, China). PCR reaction was performed using the gene-specific primers F1 (5-ATGGAATTGCCCAATATTATGCAC-3) and R1 (5-TCAGCGCAGCTTGTCGGC-3) for the presence of blaNDM. The gene sequences were confirmed, and subsequently, the NDM variant was sequenced.

2. MIC against CRKP

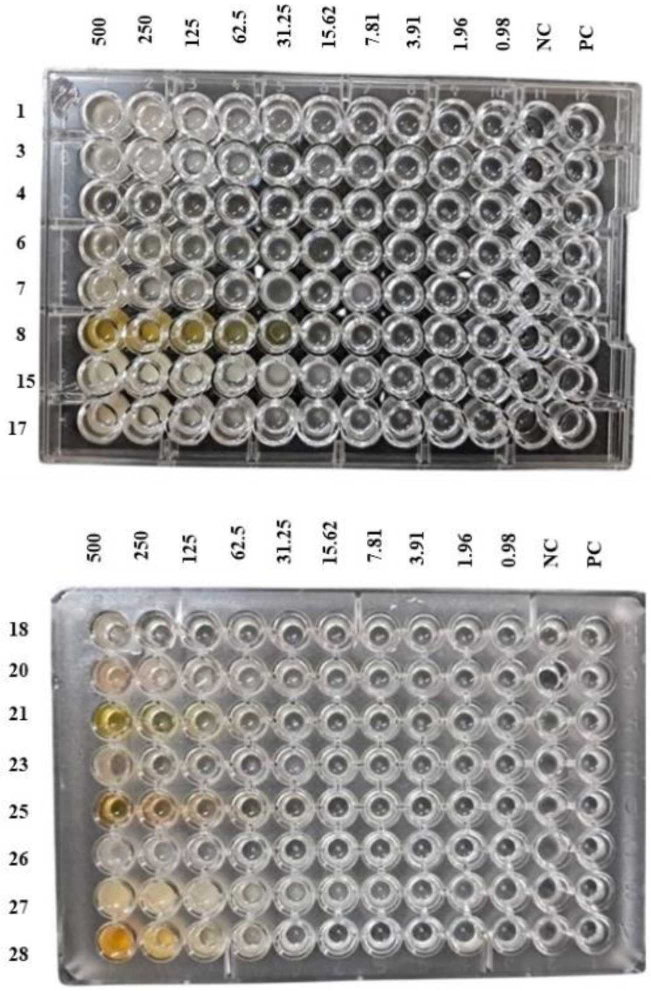

Figure S1- Minimum Inhibitory Concentration of Different Compounds Against CRKP.

3. Spectroscopic data:

5-(Isoquinolin-5-yl)thienyl-2-sulfonamide (**3a**). Yellow solid (273 mg, 94%). M.P. 236−237 ºC; **^{1}H NMR (500 MHz, acetone-d$_6$)** δ 9.40 (s, 1H), 8.61−8.59 (m, 1H), 8.24 (d, J = 8.0 Hz, 1H), 8.00 (d, J = 5.5 Hz, 1H), 7.92 (dd, J = 7.5, 1.5 Hz, 1H), 7.80−7.73 (m, 2H), 7.41−7.39 (m, 1H), 7.06 (br s, 2H); **^{13}C NMR (125.8 MHz, acetone-d$_6$)** δ 154.0, 147.0, 145.7, 145.3, 134.5, 133.2, 131.7, 130.6, 130.1, 129.9, 128.8, 127.9, 118.2.

5-(3-Cyanophenyl)thienyl-2-sulfonamide (**3b**). Yellow solid (232 mg, 88%). M.P. 168−170 ºC; **^{1}H NMR (500 MHz, DMSO-d$_6$)** δ 8.27 (s, 1H), 8.04 (d, J = 8.0 Hz, 1H), 7.86 (d, J = 8.0 Hz, 1H), 7.82 (br s, 2H), 7.67 (t, J = 8.0 Hz, 1H), 7.59 (d, J = 4.0 Hz, 1H), 7.00 (d, J = 3.5 Hz, 1H); **^{13}C NMR (125.8 MHz, DMSO-d$_6$)** δ 145.6, 145.1, 133.6, 132.2, 131.0, 130.6, 130.5, 129.2, 125.4, 118.3, 112.5.

5-(3-Hydroxyphenyl)thienyl-2-sulfonamide (**3c**). Yellow solid (240 mg, 94%). M.P. 176−178 ºC; **^{1}H NMR (500 MHz, acetone-d$_6$)** δ 7.56 (dd, J = 3.5, 1.5 Hz, 1H), 7.38 (dd, J = 4.0, 1.5 Hz, 1H), 7.31−7.24 (m, 2H), 7.20−7.14 (m, 3H), 6.89 (d, J = 8.0 Hz, 1H); **^{13}C NMR (125.8 MHz, acetone-d$_6$)** δ 158.6, 149.7, 144.6, 134.8, 132.0, 131.0, 123.7, 118.0, 116.7, 113.3.

5-(2-Formylphenyl)thienyl-2-sulfonamide (**3d**). Yellow solid (246 mg, 92%). M.P. 177−178 ºC; **^{1}H NMR (500 MHz, acetone-d$_6$)** δ 10.18 (s, 1H), 7.99 (d, J = 7.5 Hz, 1H), 7.79−7.74 (m, 1H), 7.68−7.62 (m, 3H), 7.26 (dd, J = 7.8, 3.8 Hz, 1H), 7.01 (br s, 2H); **^{13}C NMR (125.8 MHz, acetone-d$_6$)** δ 191.3, 147.7, 144.5, 136.7, 135.2, 134.7, 132.3, 131.4, 130.3, 130.2, 128.9.

5-(6-Formylbenzo[d][1,3]dioxol-5-yl)thienyl-2-sulfonamide (**3e**). Yellow solid (274 mg, 88%). M.P. 210−212 °C; **^{1}HNMR (500 MHz, acetone-d$_6$)** δ 9.94 (s, 1H), 7.63 (dd, J = 4.0, 1.5 Hz, 1H), 7.36 (s, 1H), 7.63 (dd, J = 4.0, 1.5 Hz, 1H), 7.07 (s, 1H), 6.99 (br s, 2H), 6.24 (s, 2H); **^{13}C NMR (125.8 MHz, acetone-d$_6$)** δ 189.4, 153.4, 150.2, 147.8, 144.3, 133.9, 131.4, 130.8, 130.4, 111.6, 106.9, 104.1.

5-(2-Nitropyridin-4-yl)thienyl-2-sulfonamide (**3f**). Yellow solid (223 mg, 78%). M.P. 202−203 ºC; **^{1}H NMR (500 MHz, acetone-d$_6$)** δ 9.02 (d, J = 1.5 Hz, 1H), 8.60−8.53 (m, 1H), 8.45−8.36 (m, 1H), 7.84 (d, J = 6.5 Hz, 1H), 7.71 (d, J = 6.5 Hz, 1H), 7.12 (br s, 2H); **^{13}C NMR (125.8 MHz, acetone-d$_6$)** δ 157.1, 148.7, 146.5, 143.1, 137.8, 135.3, 132.4, 128.1, 119.5.

5-(4-(Methoxymethyl)phenyl)thienyl-2-sulfonamide (**3g**). Yellow solid (260 mg, 92%). M.P. 191−192 ºC; **^{1}HNMR (500 MHz, acetone-d$_6$)** δ 7.70−7.65 (m, 2H), 7.58 (d, J = 3.5 Hz, 1H), 7.46−7.39 (m, 3H), 6.89 (br s, 2H), 4.47 (s, 2H), 3.36 (s, 3H); **^{13}C NMR (125.8 MHz, acetone-d$_6$)** δ 149.8, 145.0, 140.8, 133.1, 132.3, 129.2, 126.8, 124.0, 74.4, 58.3.

[2,3'-Bithienyl]-5-sulfonamide (**3h**). Brown solid (240 mg, 98%). M.P. 181−182 ºC; **^{1}H NMR (500 MHz, acetone-d$_6$)** δ 7.81−7.79 (m, 1H), 7.62 (dd, J = 5.0, 3.0 Hz, 1H), 5.54 (d, J = 4.0 Hz, 1H), 7.46 (dd, J = 5.0, 1.0 Hz, 1H); **^{13}C NMR (125.8 MHz, acetone-d$_6$)** δ 144.8, 144.0, 135.1, 132.1, 128.4, 126.7, 124.0, 122.7.

N-(3-(5-Sulfamoylthiophen-2-yl)phenyl)acetamide (**3i**). Yellow solid (172 mg, 58%). M.P. 209−210 ºC; **^{1}H NMR (500 MHz, acetone-d$_6$)** δ 9.29 (s, 1H), 8.15 (s, 1H), 7.63−7.58 (m, 2H), 7.44−7.38 (m, 3H), 6.92 (br s, 2H), 2.13 (s, 3H); **^{13}C NMR (125.8 MHz, acetone-d$_6$)** δ 169.3, 149.9, 145.1, 141.4, 134.3, 132.2, 130.5, 124.0, 121.5, 120.2, 117.3, 24.4.

5-(1-(Phenylsulfonyl)-3a,7a-dihydro-1H-indol-3-yl)thienyl-2- sulfonamide (**3j**). Yellow solid (326 mg, 78%). M.P. 246−248 ºC; **^{1}H NMR (500 MHz, acetone-d$_6$)** δ 8.20 (s, 1H), 8.13 (d, J = 7.0 Hz, 1H), 7.92 (d, J = 7.5 Hz, 1H), 7.70 (t, J = 6.5 Hz, 1H), 7.65 (d, J = 4.0 Hz, 1H), 7.63−7.59 (m, 2H), 7.53 (d, J = 4.0 Hz, 1H), 7.48 (t, J = 8.0 Hz, 1H), 7.43−7.39 (m, 1H); **^{13}C NMR (125.8 MHz, acetone-d$_6$)** δ 144.7, 140.3, 138.4, 136.1, 135.4, 131.8, 130.5, 128.7, 127.9, 126.5, 125.6, 125.1, 121.1, 116.8, 114.6.

5'-Formyl-3'-methyl-[2,2'-bithienyl]-5-sulfonamide (**3k**). Yellow solid (224 mg, 78%). M.P. 188−189 ºC; **^{1}H NMR (500 MHz, acetone-d$_6$)** δ 9.92 (s, 1H), 7.85 (s, 1H), 7.64 (d, J = 7.0 Hz, 1H), 7.43 (d, J = 4.0 Hz, 1H), 7.02 (br s, 2H), 2.80 (s, 3H); **^{13}C NMR (125.8 MHz, acetone-d$_6$)** δ 183.9, 147.5, 142.5, 141.7, 140.6, 139.1, 137.9, 131.9, 128.1, 15.8.

5-(4-(tert-Butyl)phenyl)thienyl-2-sulfonamide (**3l**). Yellow solid (280 mg, 95%). M.P. 188−190 ºC; **1H NMR (500 MHz, acetone-d$_6$)** δ 7.68−7.50 (m, 5H), 7.42 (d, J = 4 Hz, 1H), 6.90 (br s, 2H), 1.36 (s, 9H); **^{13}C NMR (125.8 MHz, acetone-d$_6$)** δ 152.8, 149.9, 144.4, 132.1, 130.9, 126.9, 126.5, 123.4, 35.1, 31.7, 31.3.

5-(Furan-3-yl)thienyl-2-sulfonamide (**3m**). Brown solid (179 mg, 78%). M.P. 186−187 ºC; **^{1}H NMR (500 MHz, acetone-d$_6$)** δ 8.04−8.01 (m, 1H), 7.67−7.63 (m, 1H), 7.53−7.49 (m, 1H), 7.23−7.19 (m, 1H), 6.87 (br s, 2H), 6.80 (s, 1H); **^{13}C NMR (125.8 MHz, acetone-d$_6$)** δ 145.2, 143.5, 140.4, 131.7, 129.3, 124.0, 120.2, 109.6.

5-(2-(Benzyloxy)phenyl)thienyl-2-sulfonamide (**3n**). Yellow solid (235 mg, 68%). M.P. 262−264 ºC; **^{1}H NMR (500 MHz, acetone-d$_6$)** δ 7.89 (dd, J = 7.5, 1.5 Hz, 1H), 7.68−7.60 (m, 4H), 7.51−7.46 (m, 2H), 7.45−7.39 (m, 2H), 7.32 (d, J = 6.5 Hz, 1H), 7.17−7.11 (m, 1H), 6.90 (br s, 2H), 5.45 (s, 2H); **^{13}C NMR (125.8 MHz, acetone-d$_6$)** δ 155.4, 145.3, 144.9, 137.6, 130.6, 129.3, 129.1, 128.8, 128.4, 125.4, 122.7, 122.1, 114.3, 71.1.

5-(2-Formyl-5-methoxyphenyl)thienyl-2-sulfonamide (**3o**). Yellow solid (268 mg, 90%). M.P. 208−209 ºC; **^{1}H NMR (500 MHz, acetone-d$_6$)** δ 10.06 (s, 1H), 8.01 (d, J = 9.0 Hz, 1H), 7.68 (d, J = 3.5 Hz, 1H), 7.29 (d, J = 4.0 Hz, 1H), 7.23 (dd, J = 9.0, 2.5 Hz, 1H), 7.13 (d, J = 2.0 Hz, 1H), 7.03 (br s, 2H), 4.02 (s, 3H); **^{13}C NMR (125.8 MHz, acetone-d$_6$)** δ 189.7, 164.7, 147.8, 144.5, 139.2, 131.4, 130.2, 130.2, 128.7, 116.9, 116.4, 59.5.

5-(5-Methylfuran-2-yl)thienyl-2-sulfonamide (**3p**). Brown solid (234 mg, 96%). M.P. 192−193 ºC; **^{1}H NMR (500 MHz, acetone-d$_6$)** δ 7.52 (d, J = 4.0 Hz, 1H), 7.21 (d, J = 4.0 Hz, 1H), 6.88 (br s, 2H), 6.71 (d, J = 3.5 Hz, 1H), 6.19−6.17 (m, 1H), 2.33 (s, 3H); **^{13}C NMR (125.8 MHz, acetone-d$_6$)** δ 153.6, 146.6, 142.8, 139.0, 131.7, 121.5, 109.1, 109.0, 13.1.

4. References:

1. I. Lewis and S. James, *Performance standards for antimicrobial susceptibility testing*, 33rd Edition, M100; 2023.
2. M. U. Qamar, T. R. Walsh, M. A. Toleman, J. M. Tyrrell, S. Saleem, A. Aboklaish and S. Jahan, *Future Microbiol.*, 2019, **14**, 691-704.
3. M. U. Qamar, S. Saleem, M. A. Toleman, M. Saqalein, M. Waseem, M. A. Nisar, M. Khurshid, Z. Taj and S. Jahan, *Future Microbiol.*, 2018, **13**, 13-26.

5. NMR Spectra:

5-(Isoquinolin-5-yl)thienyl-2-sulfonamide (**3a**)

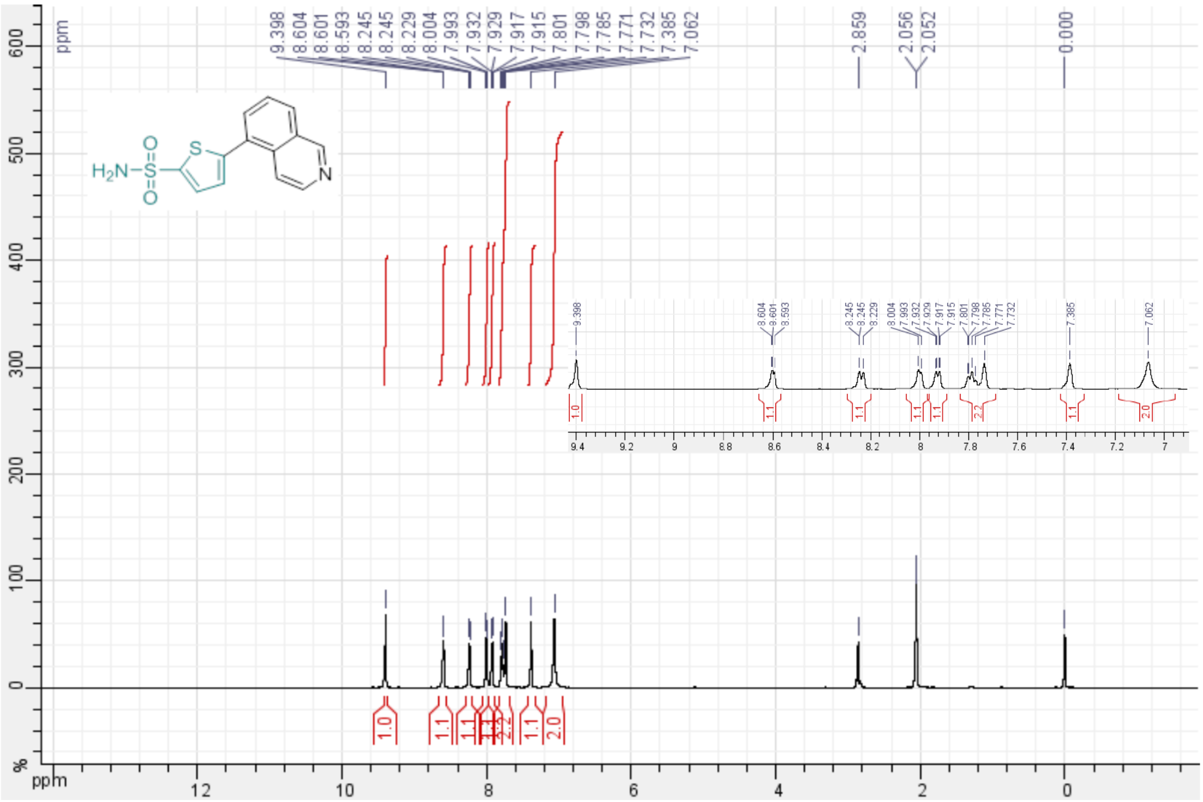

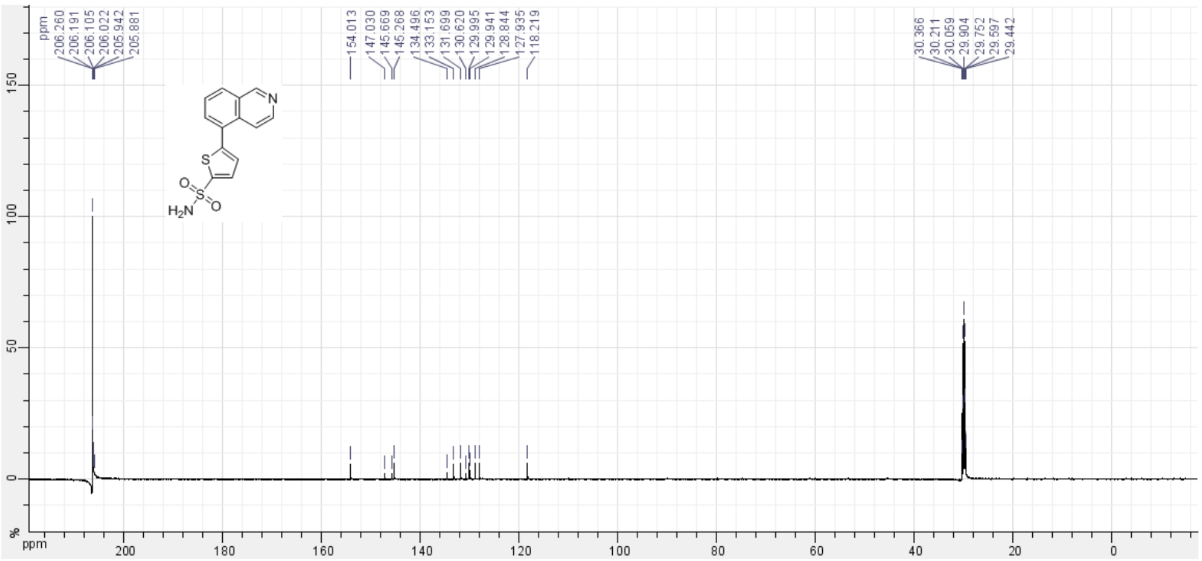

5-(3-Cyanophenyl)thienyl-2-sulfonamide **(3b)**

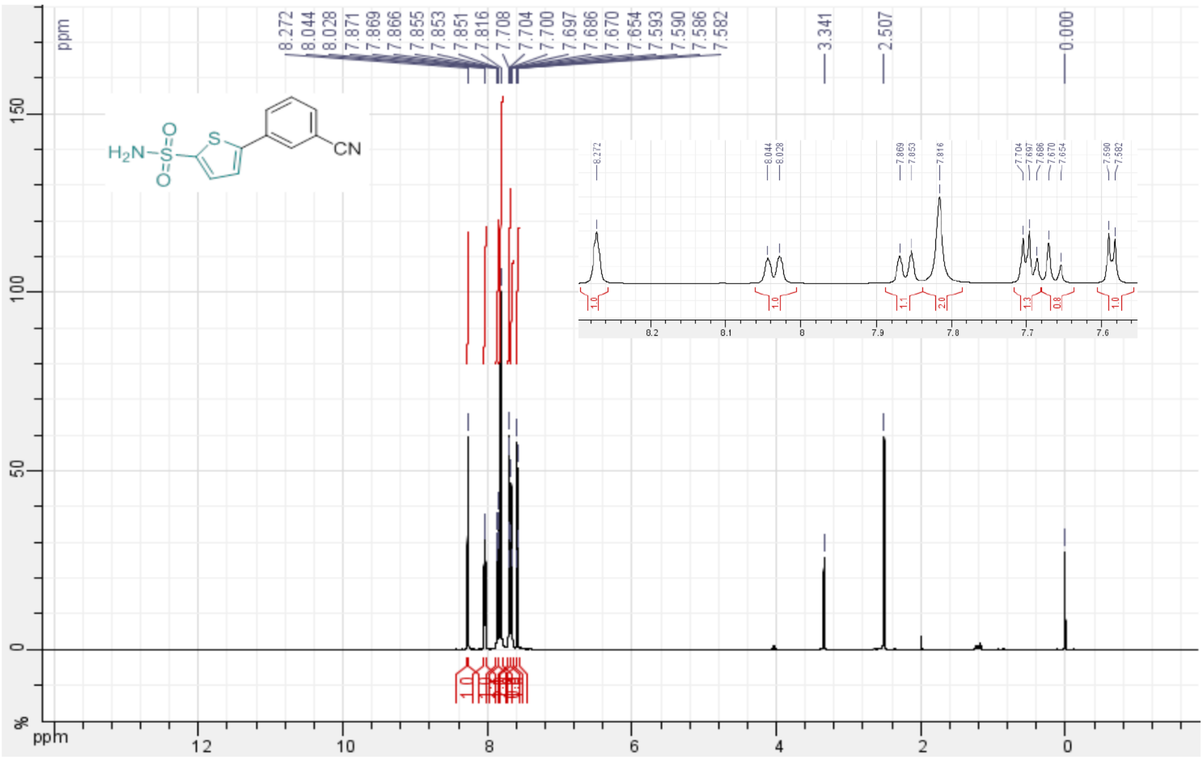

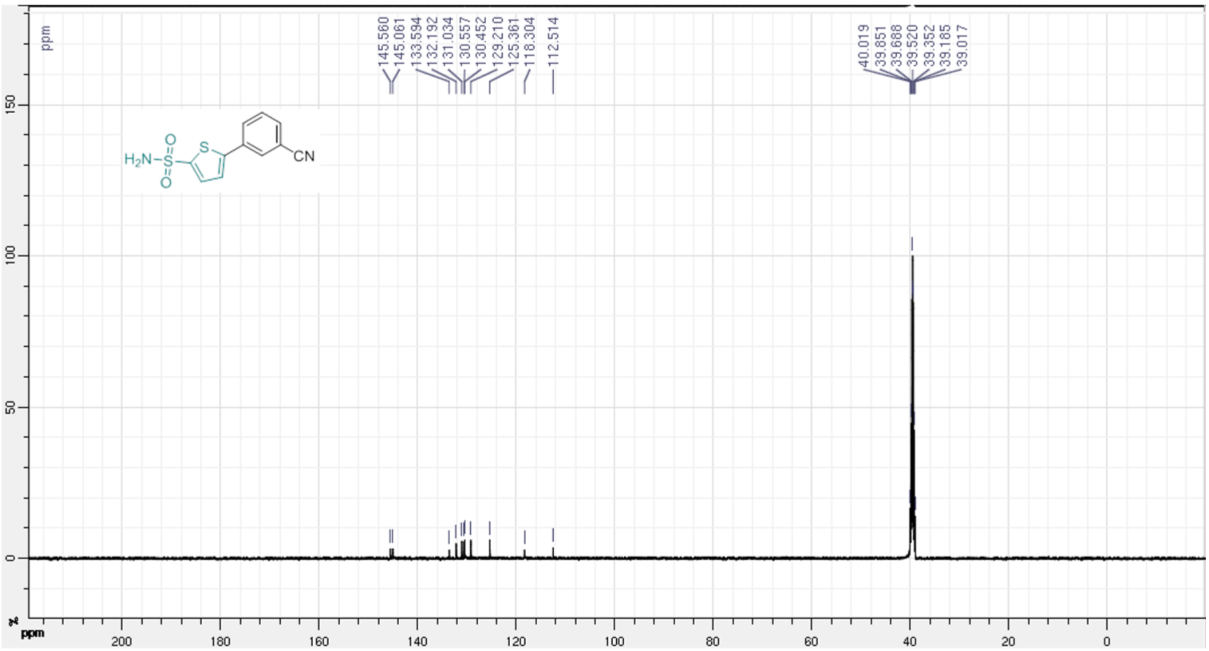

5-(3-Hydroxyphenyl)thiophene-2-sulfonamide (**3c**)

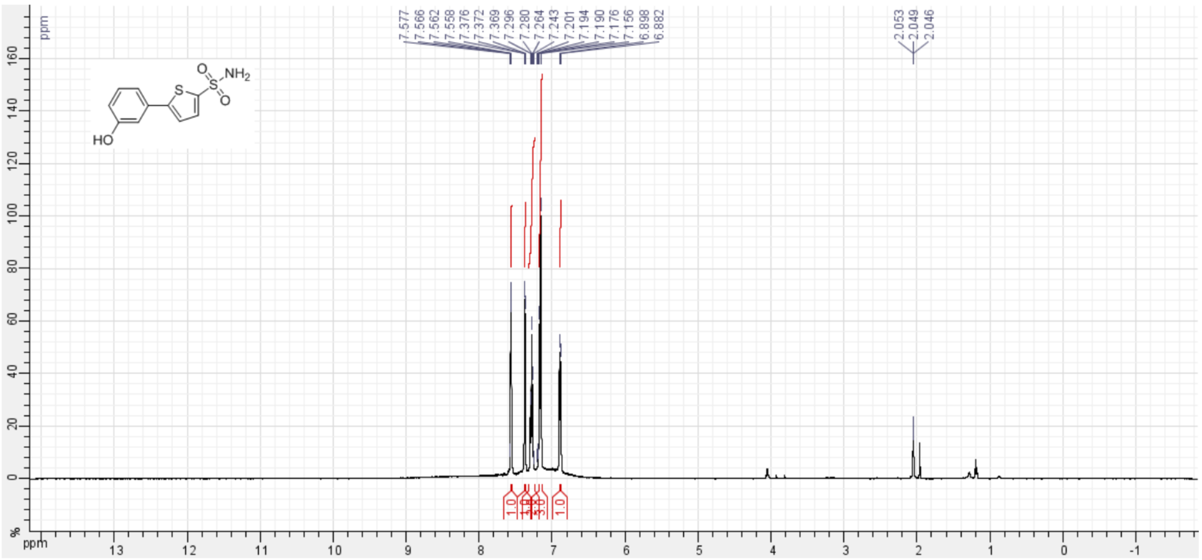

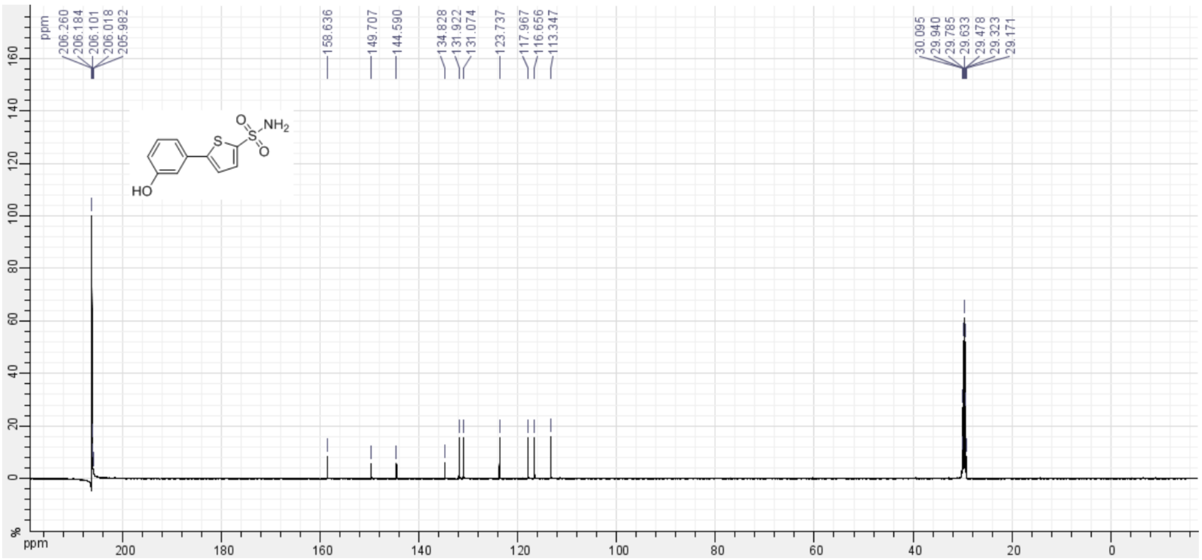

5-(2-Formylphenyl)thiophene-2-sulfonamide (**3d**)

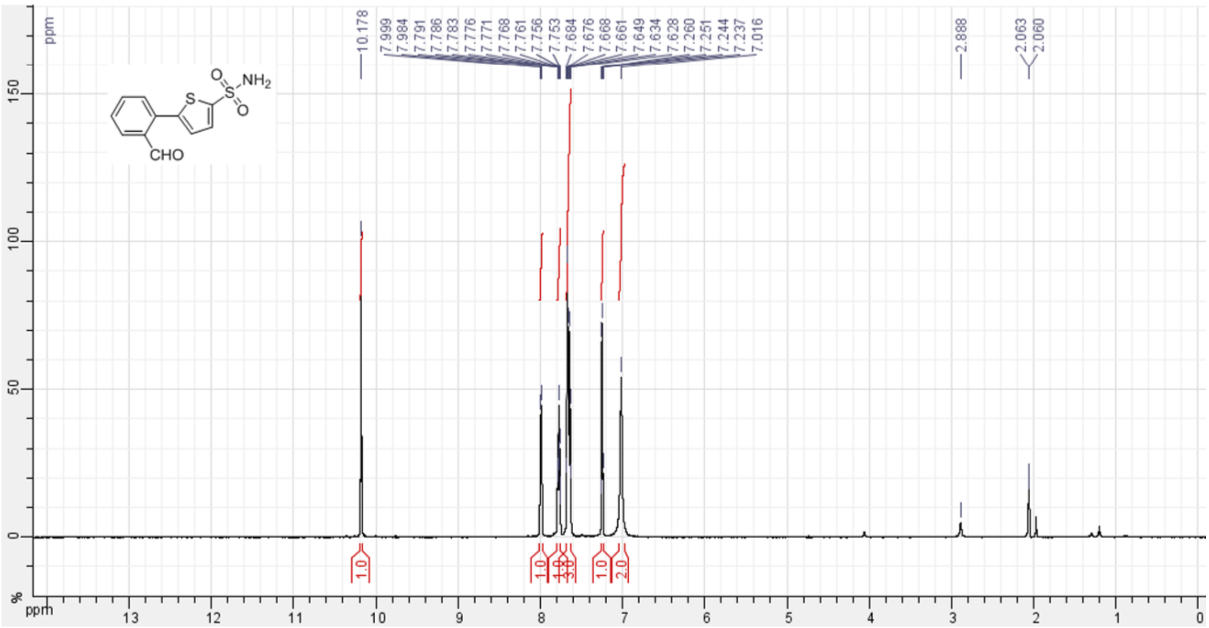

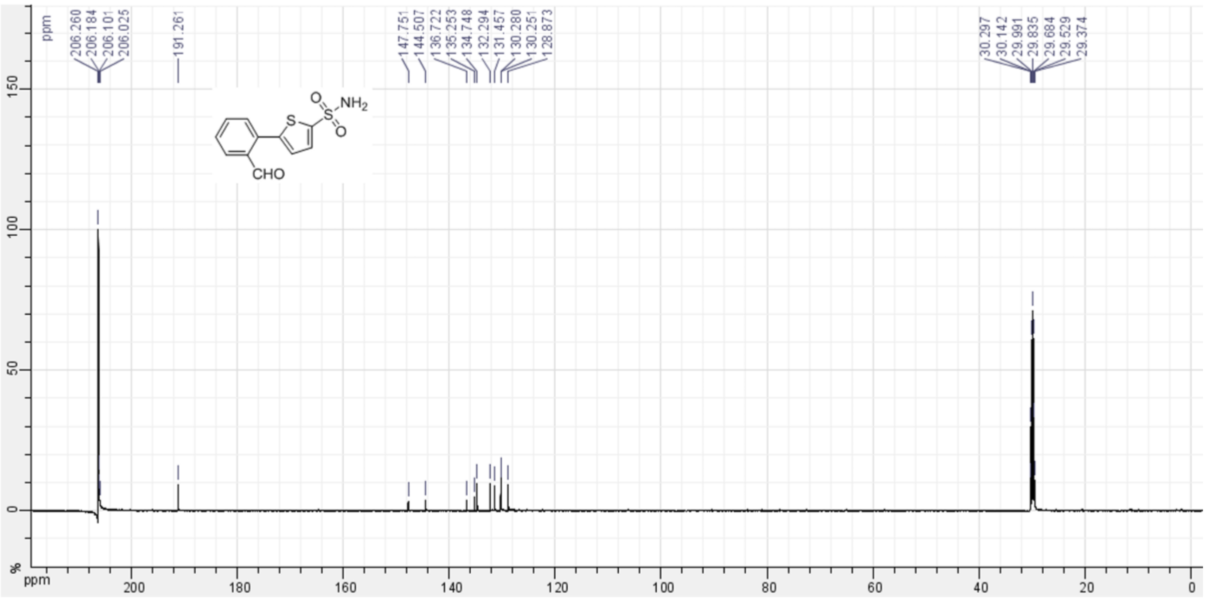

5-(6-Formylbenzo[d][1,3]dioxol-5-yl)thienyl-2-sulfonamide sulfonamide (**3e**)

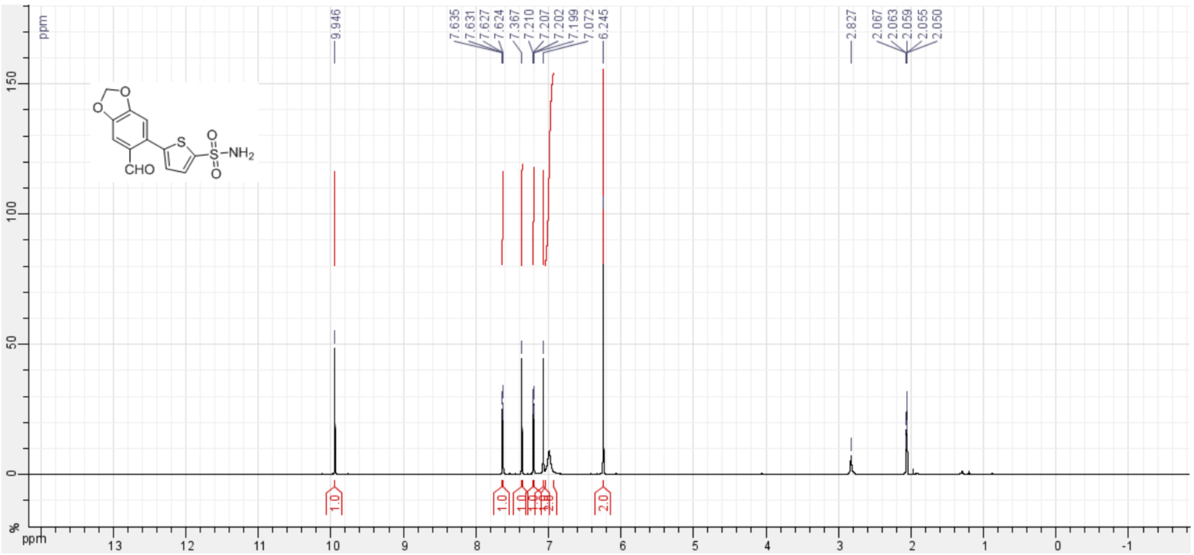

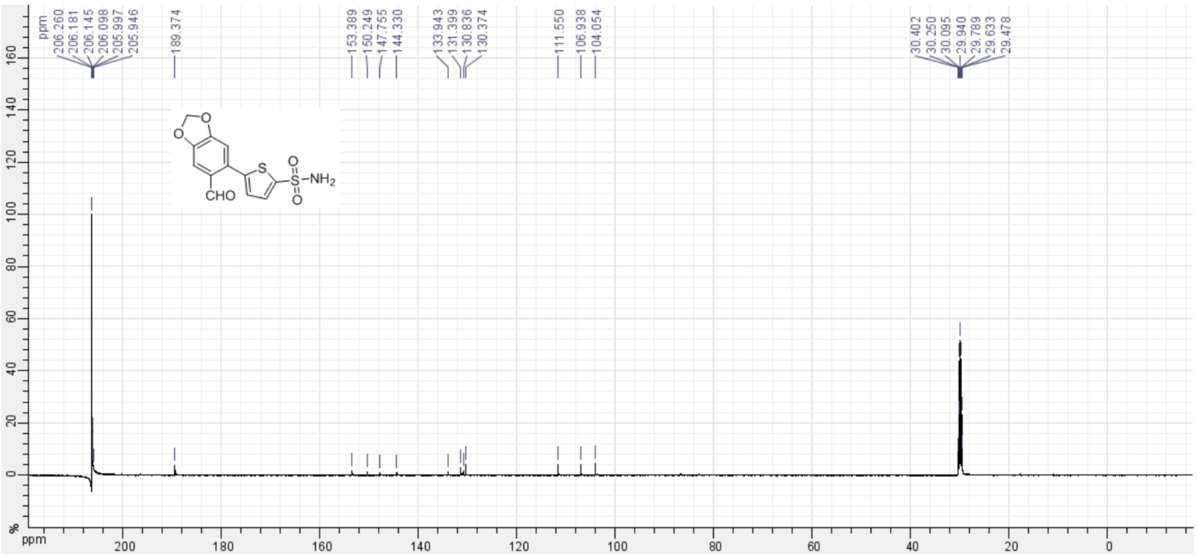

5-(2-Nitropyridin-4-yl)thienyl-2-sulfonamide **(3f)**

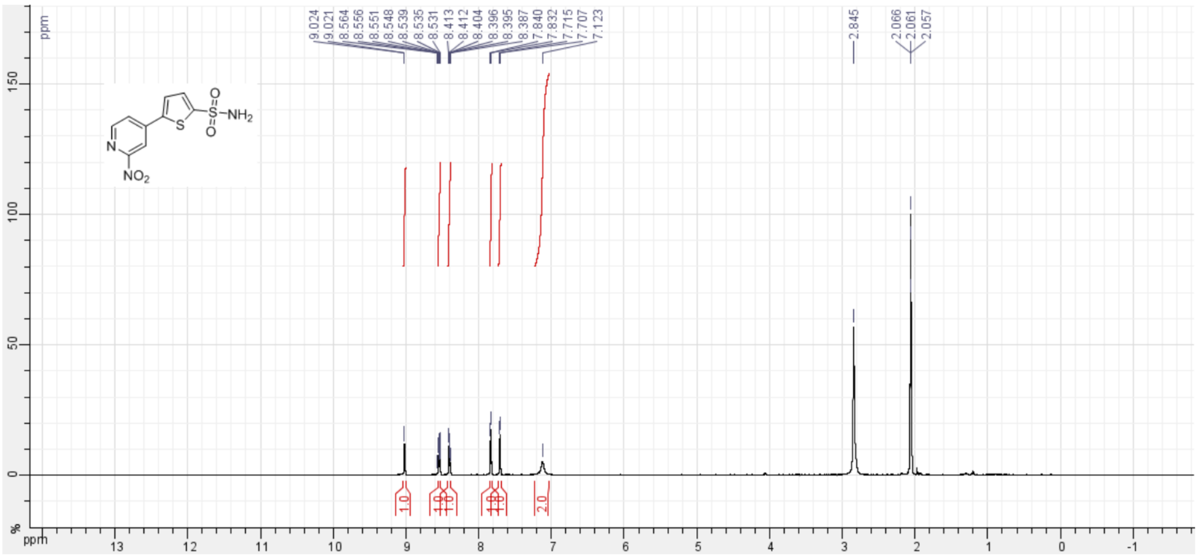

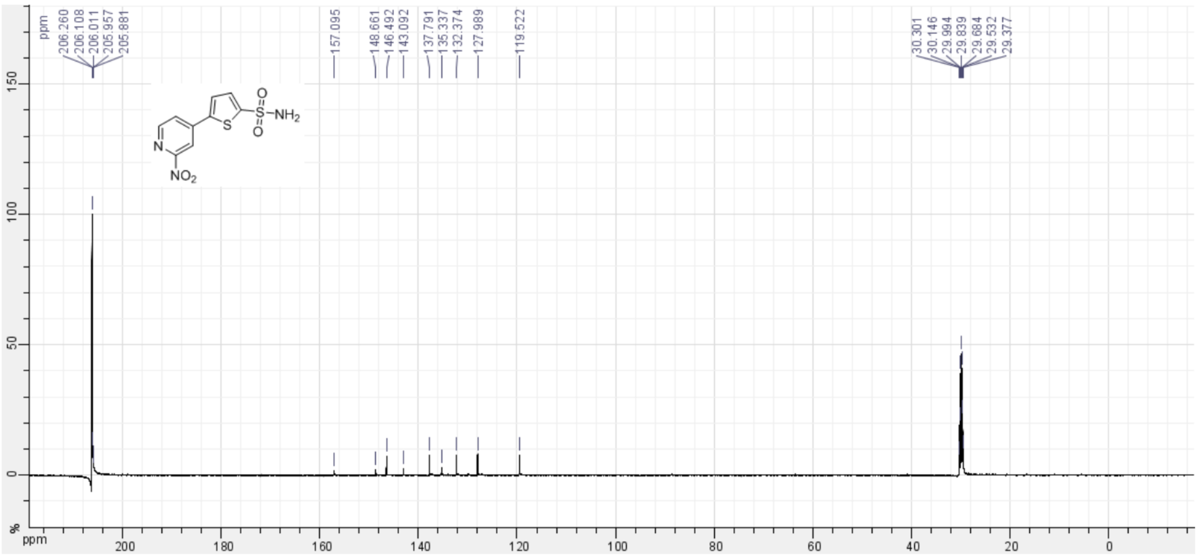

5-(4-(Methoxymethyl)phenyl)thienyl-2-sulfonamide (**3g**)

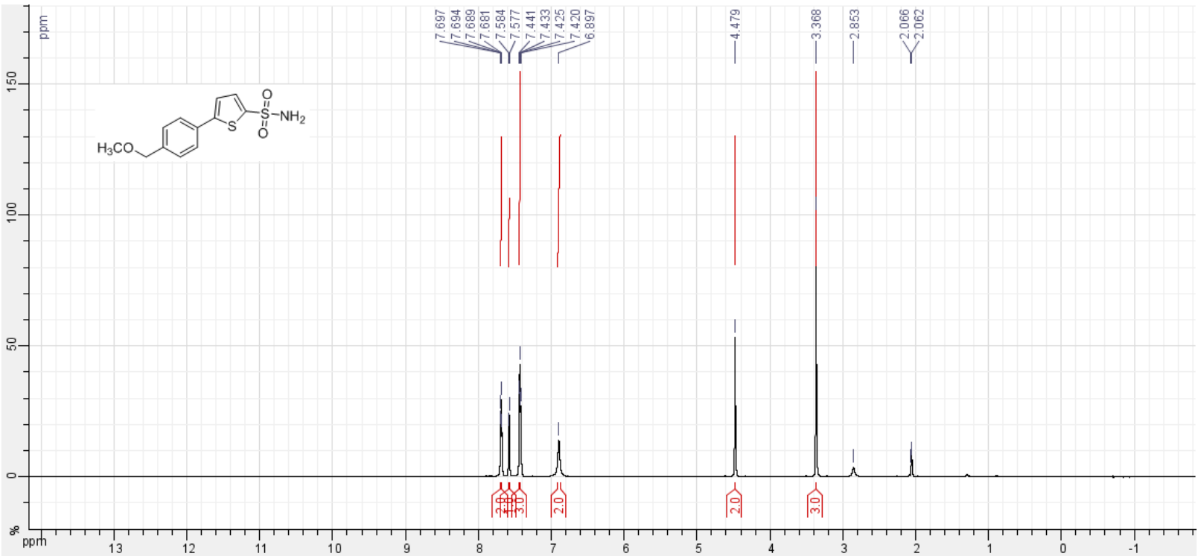

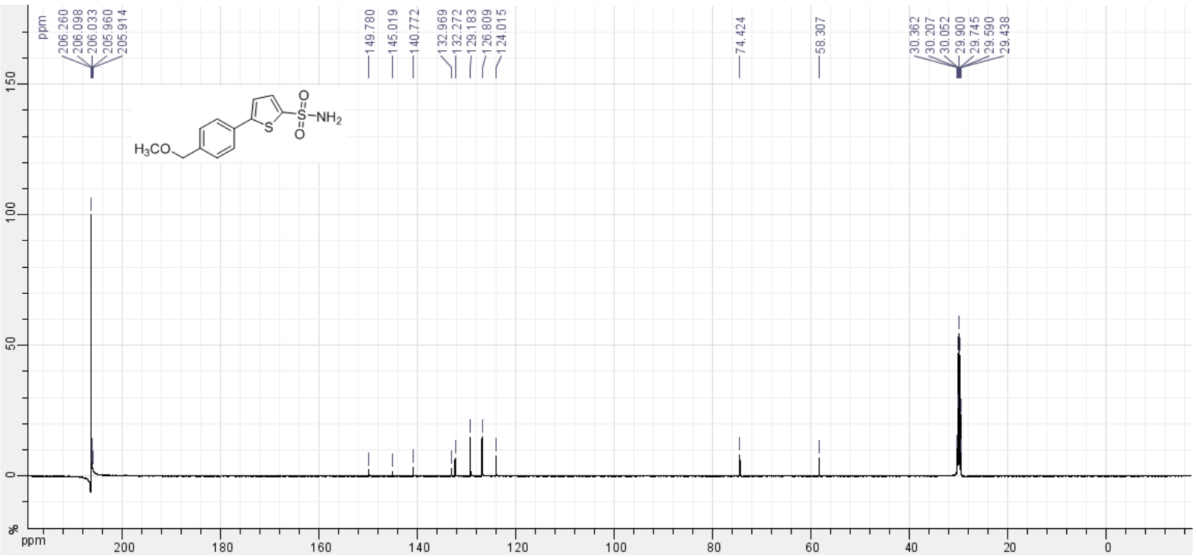

[2,3’-Bithienyl]-5-sulfonamide (**3h**)

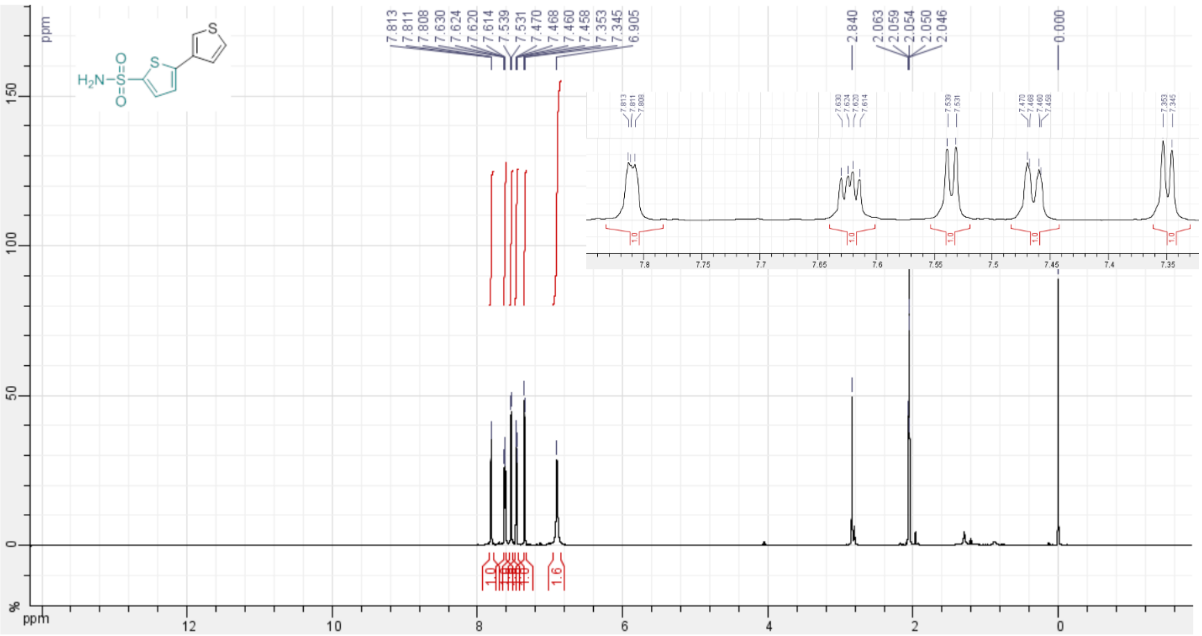

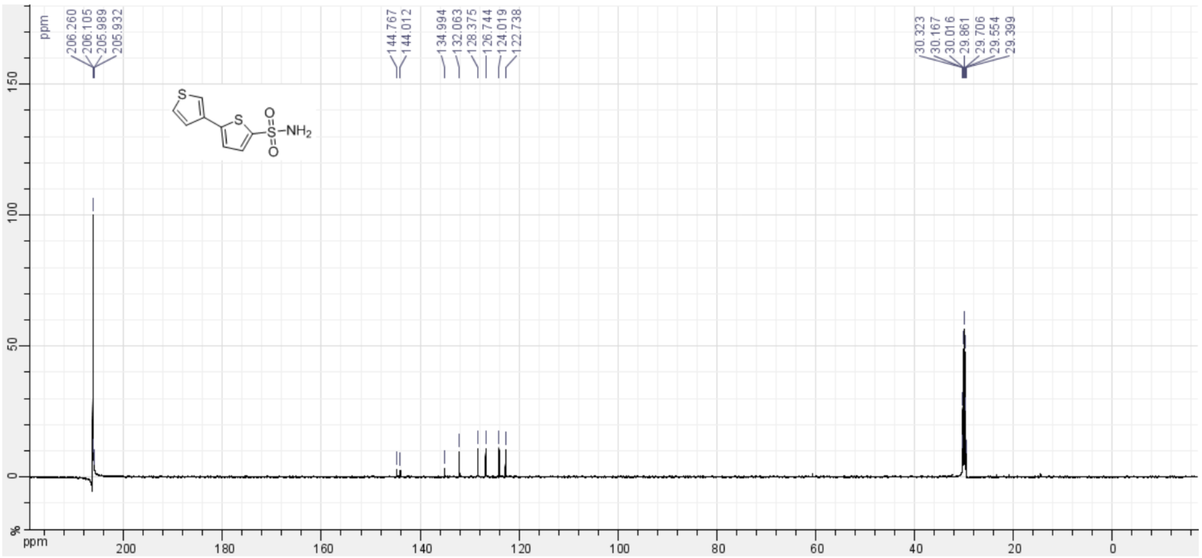

N-(3-(5-Sulfamoylthiophen-2-yl)phenyl)acetamide **(3i)**

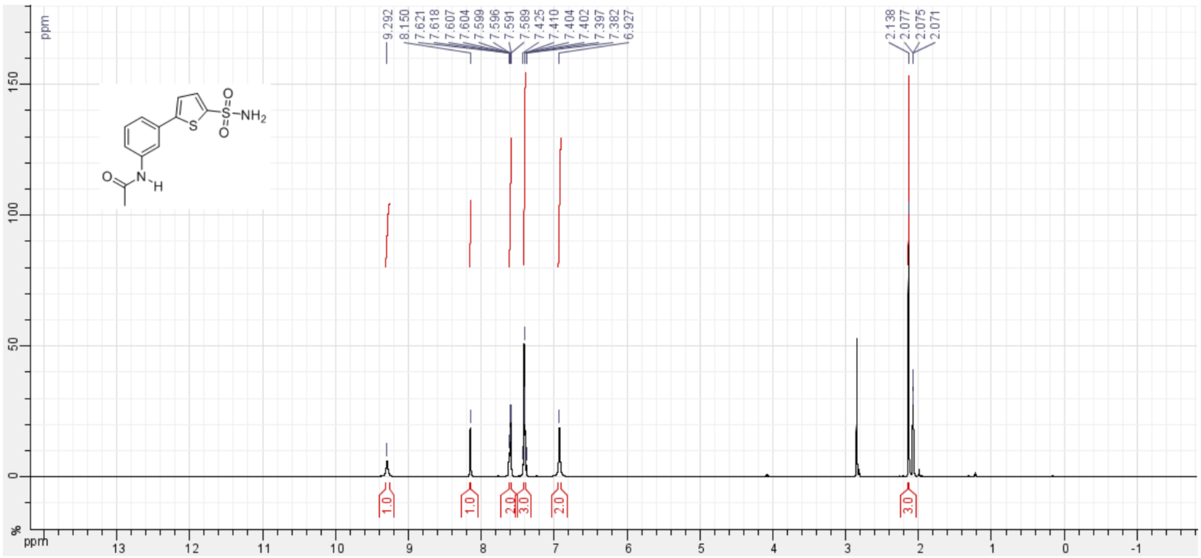

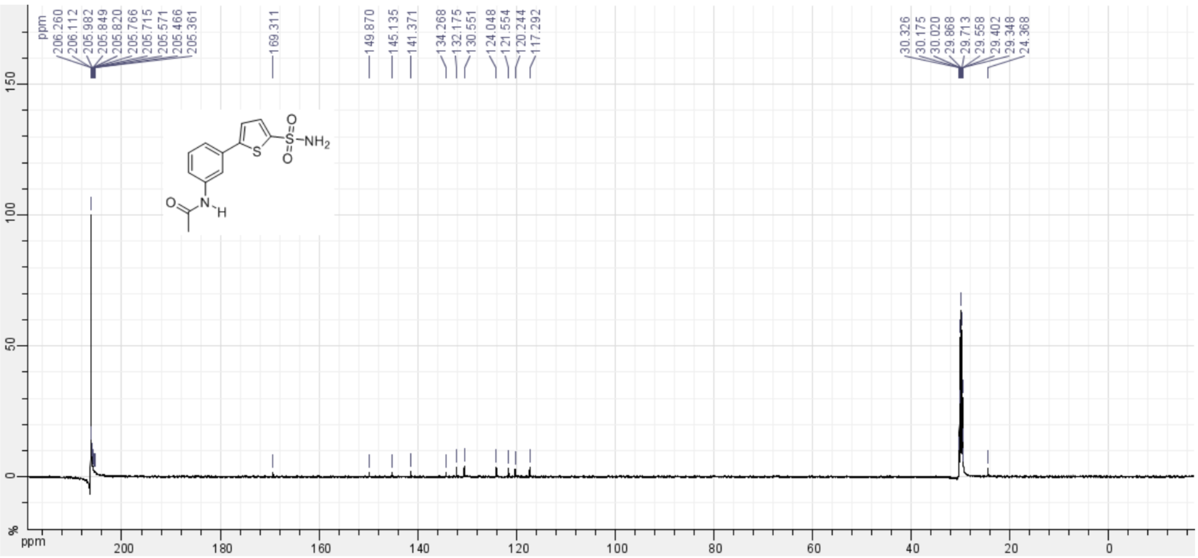

5-(1-(Phenylsulfonyl)-3a,7a-dihydro-1H-indol-3-yl)thienyl-2- sulfonamide (**3j**)

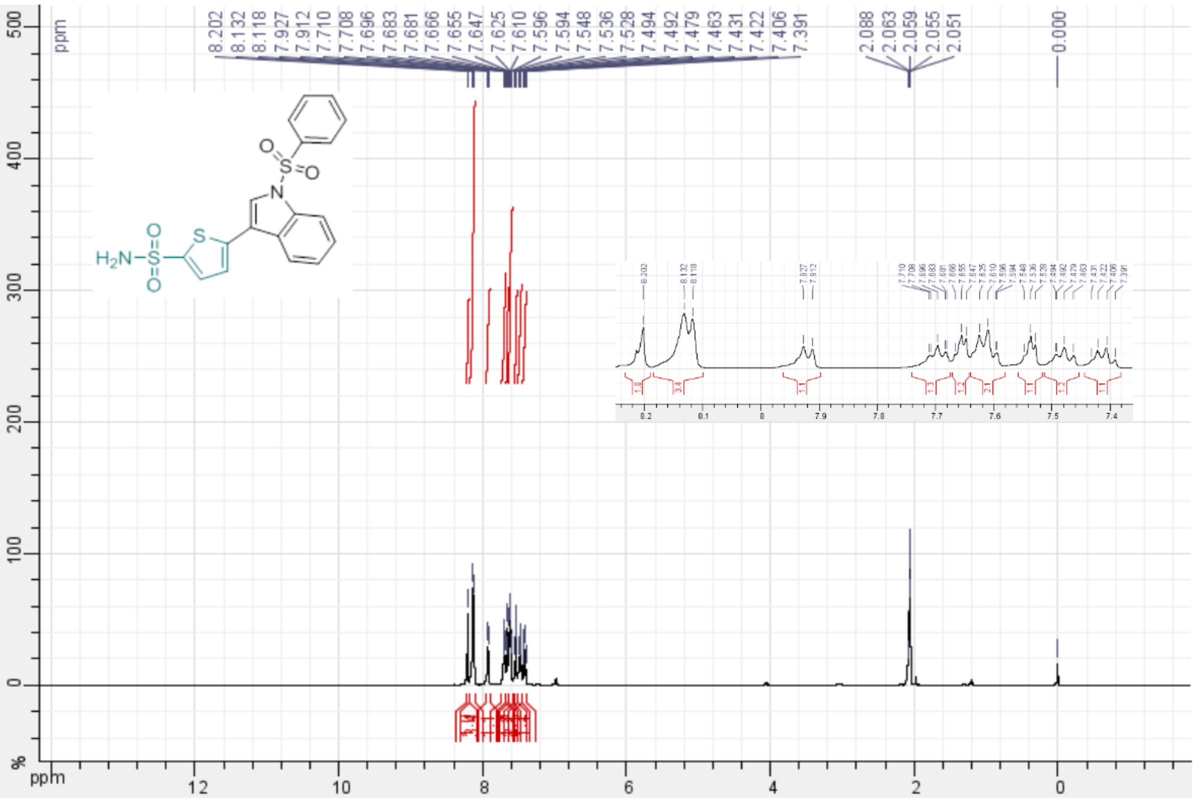

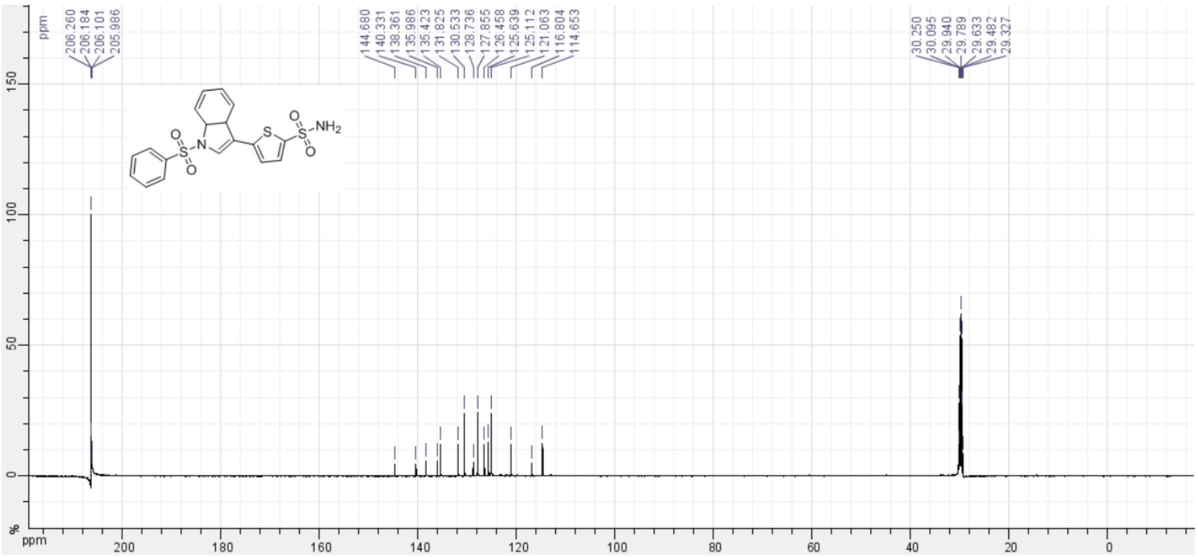

5’ 3’ [2,2’ -Formyl-methyl-bithienyl]-5-sulfonamide (**3k**)

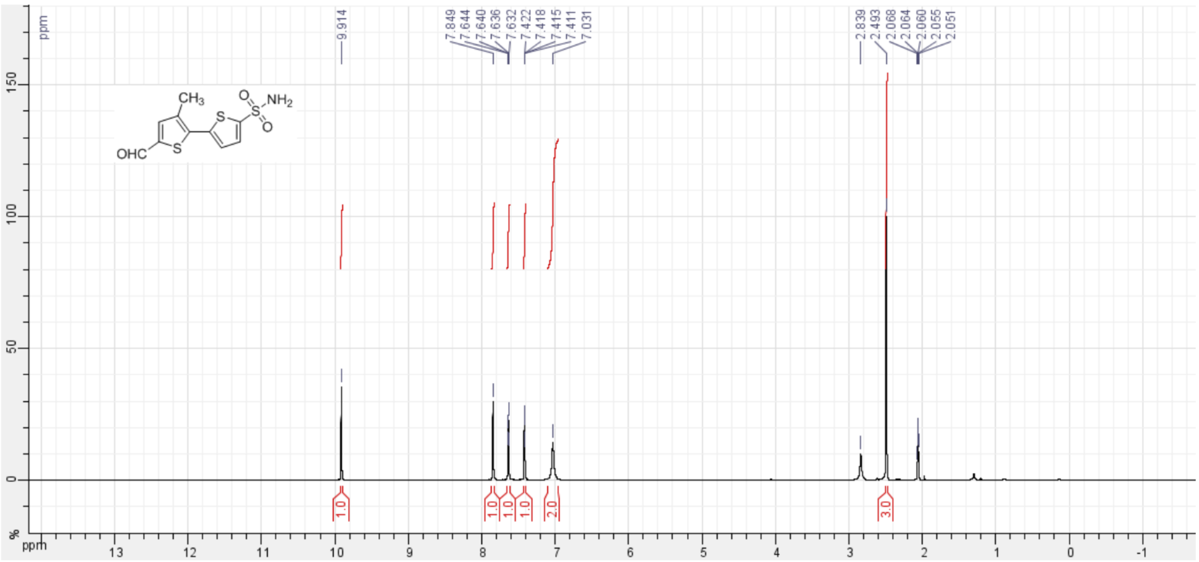

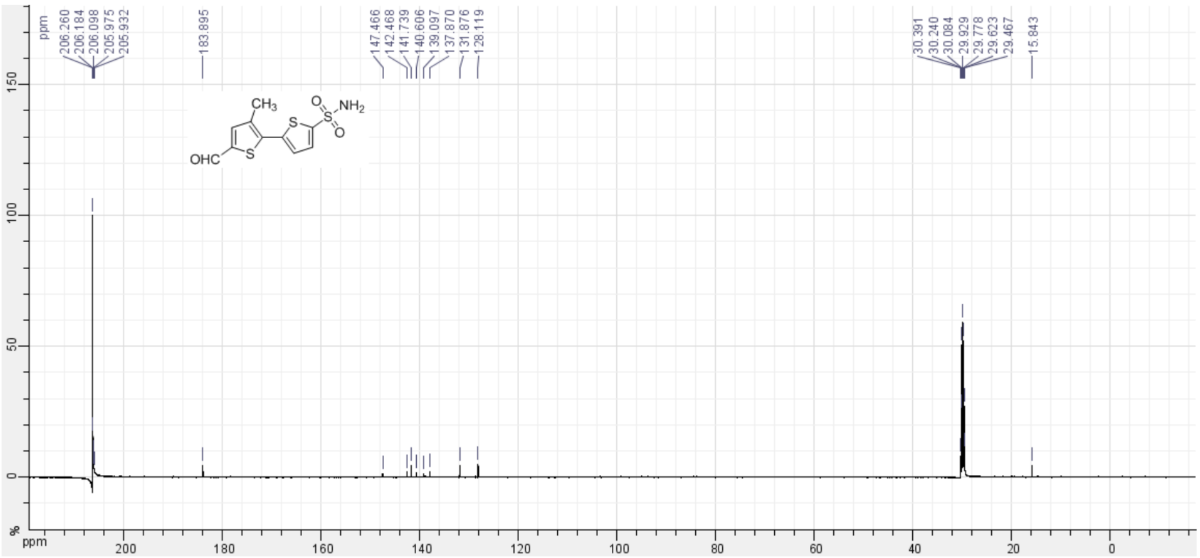

5-(4-(tert-Butyl)phenyl)thienyl-2-sulfonamide **(3l)**

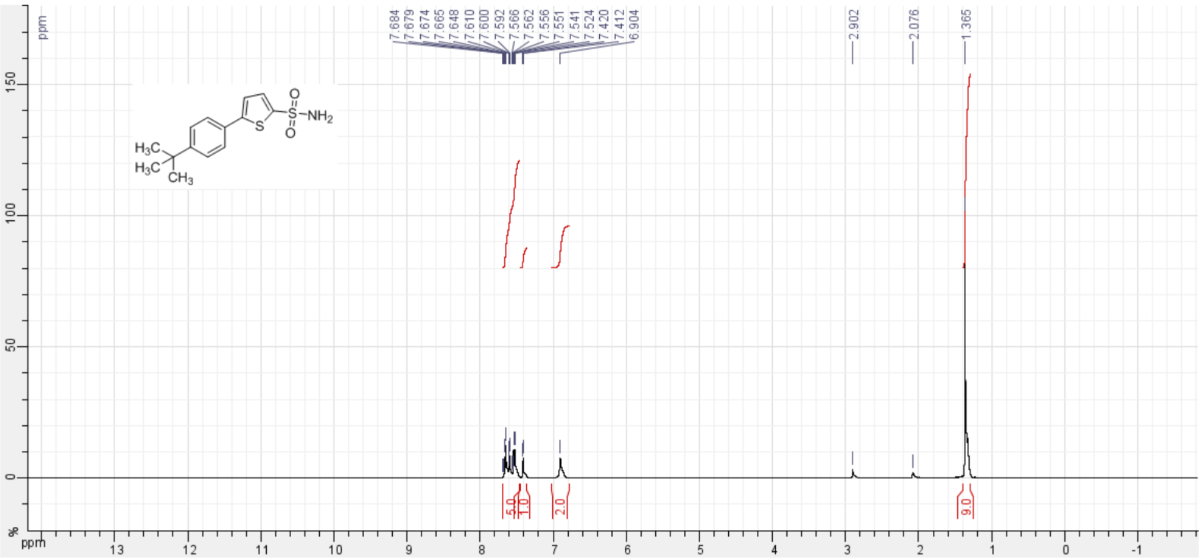

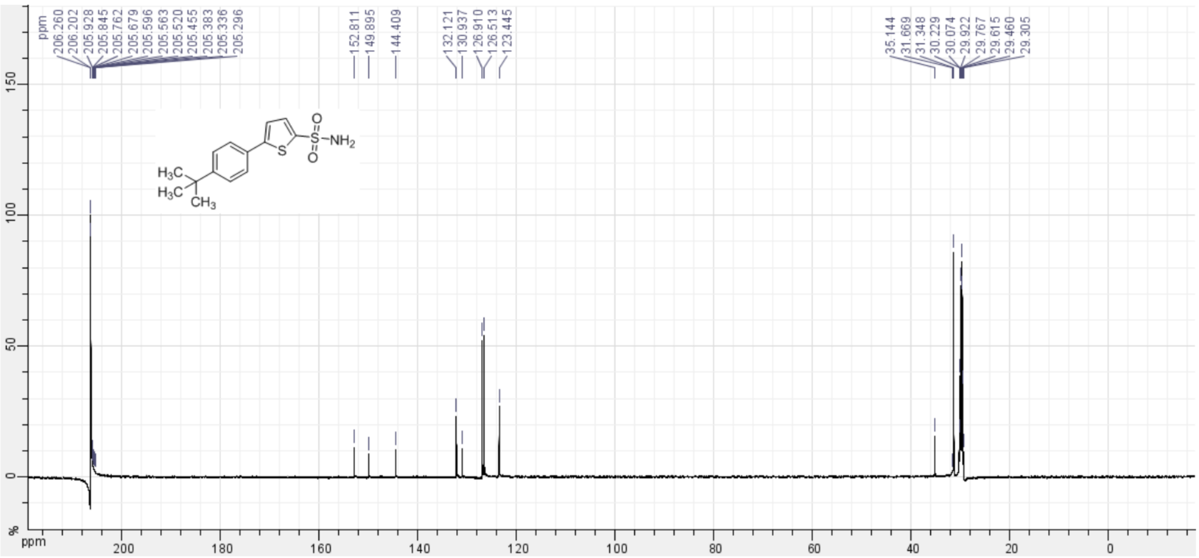

5-(Furan-3-yl)thienyl-2-sulfonamide **(3m)**

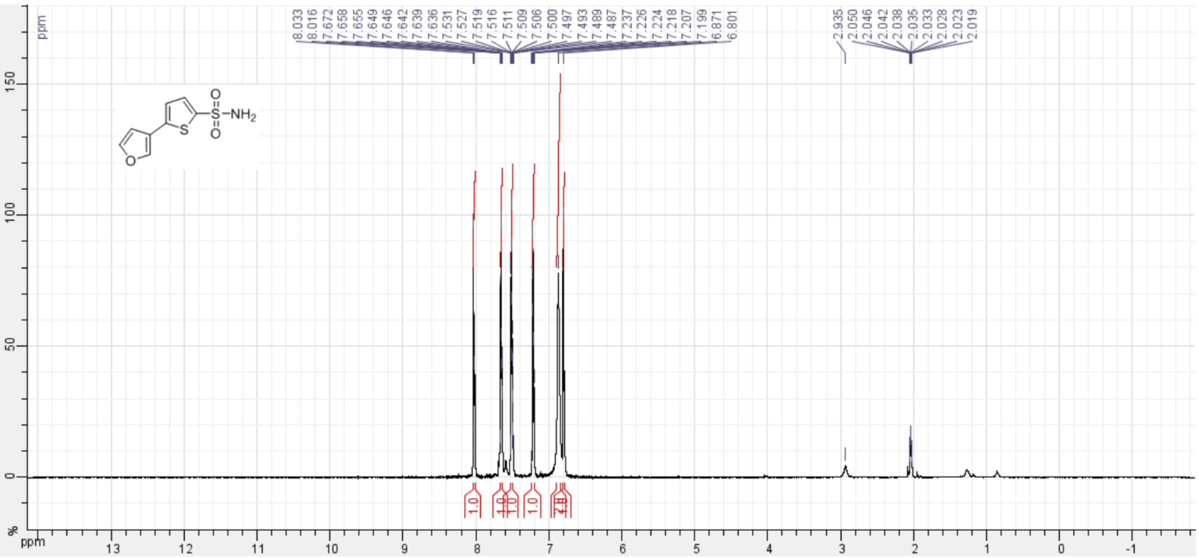

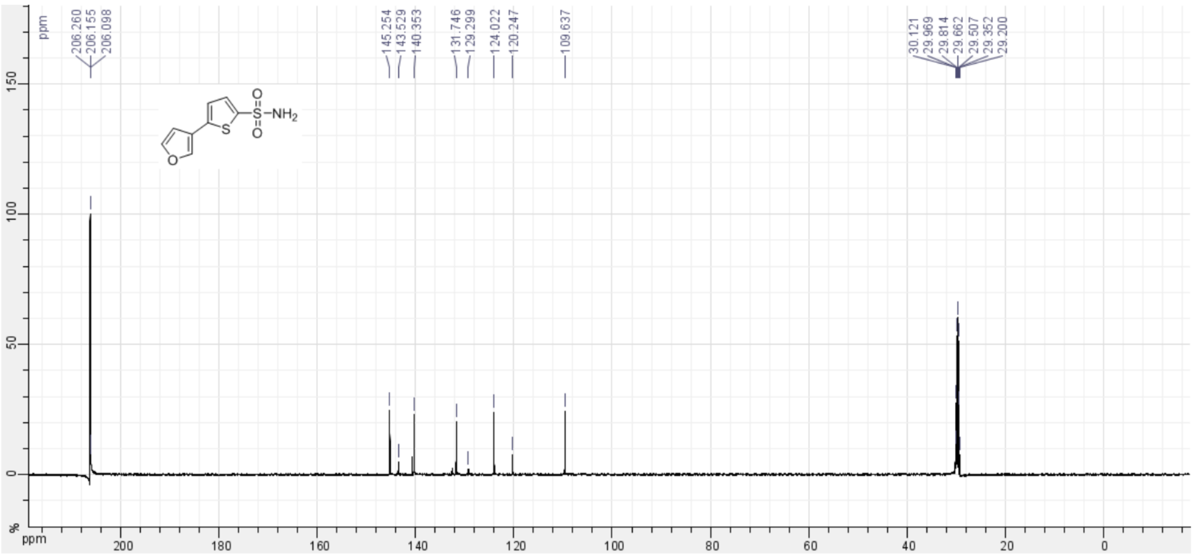

5-(2-(Benzyloxy)phenyl)thienyl-2-sulfonamide **(3n)**

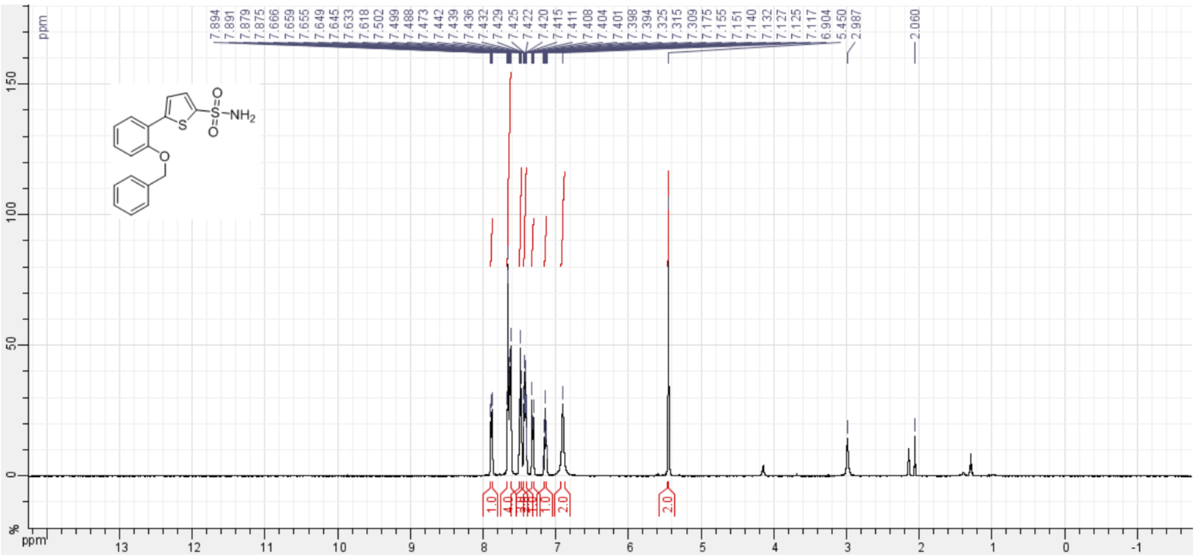

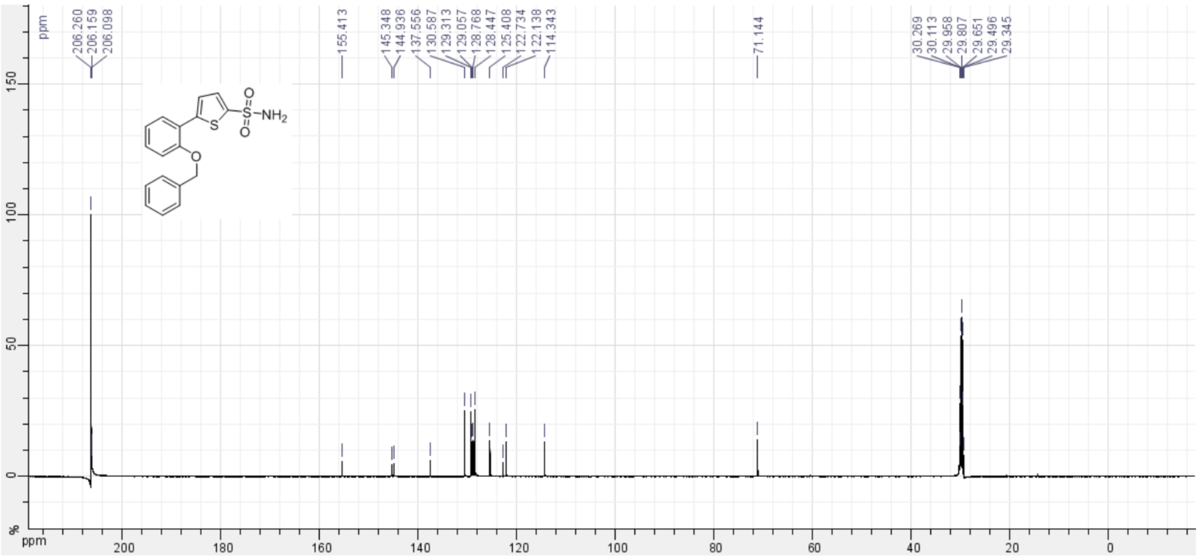

5-(2-Formyl-5-methoxyphenyl)thienyl-2-sulfonamide **(3o)**

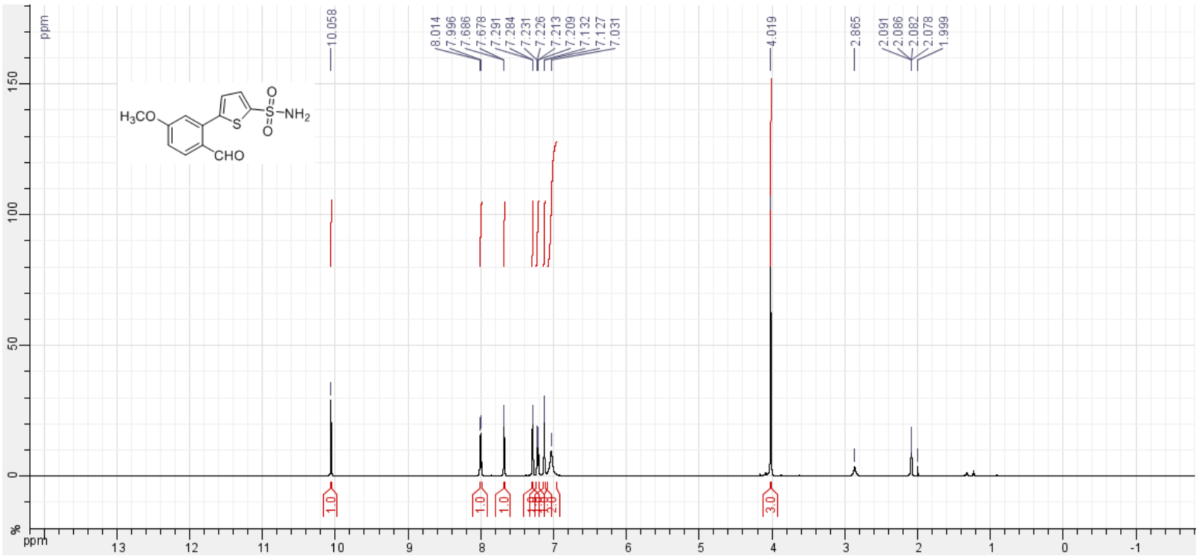

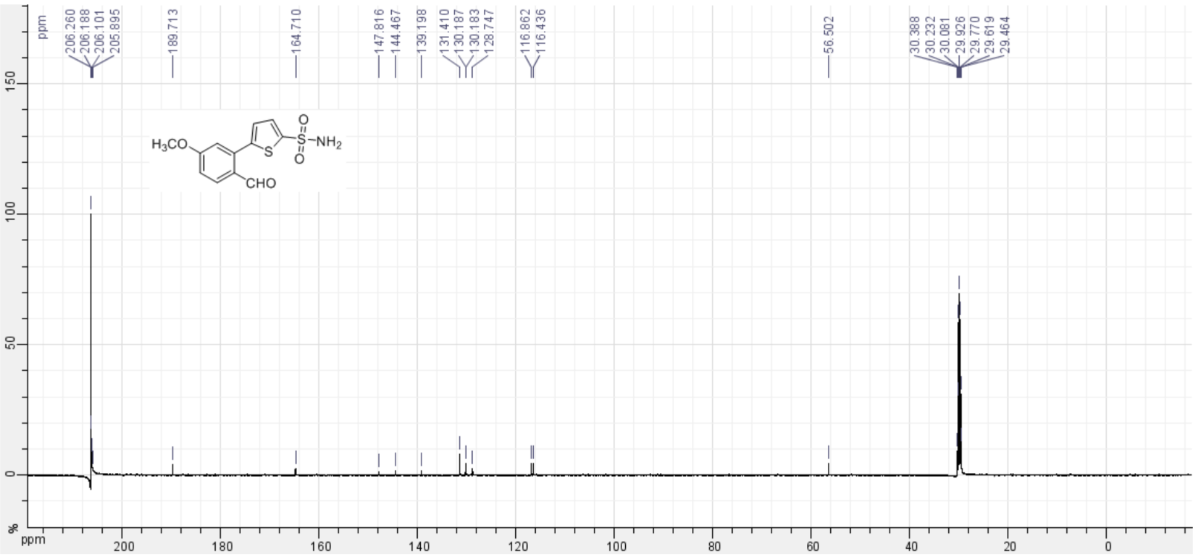

5-(5-Methylfuran-2-yl)thienyl-2-sulfonamide **(3p)**

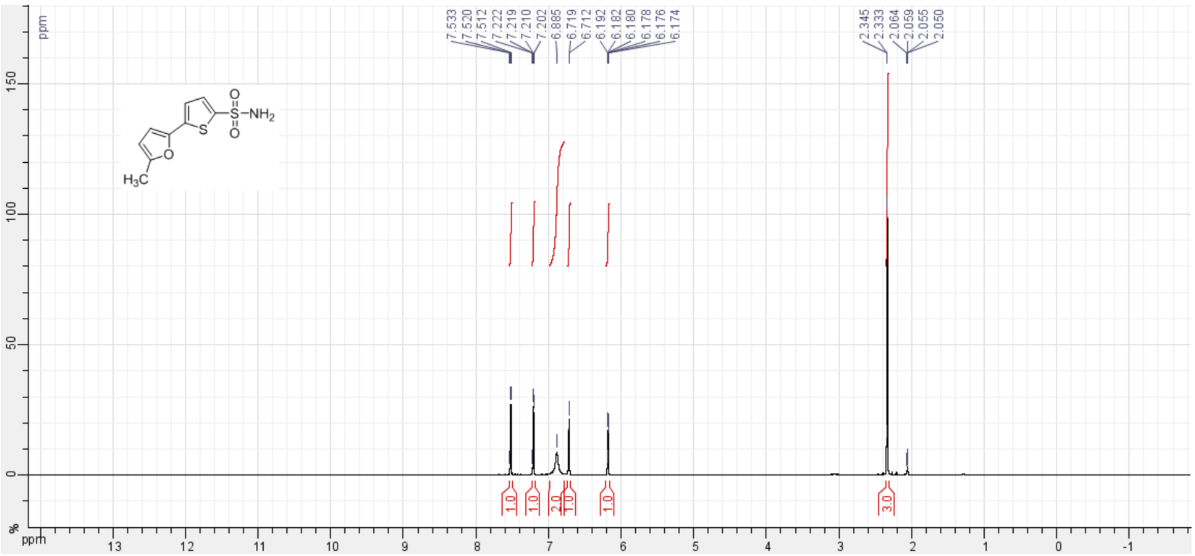

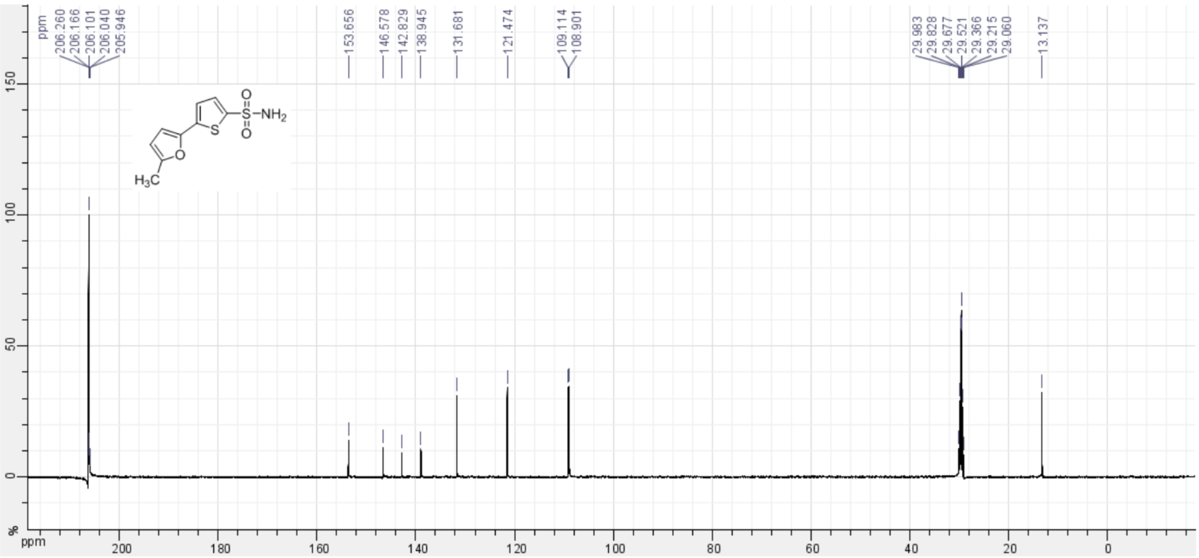
ppm
206.260
206.166
206.101
206.040
205.946
153.656
146.578
142.829
138.945
131.681
121.474
109.114
108.901
29.983
29.828
29.677
29.521
29.366
29.215
29.060
13.137
O
S
NH2
O
S
O
H3C
150
100
50
0
%
ppm
200
180
160
140
120
100
80
60
40
20
0
